# Sub-picosecond, strain-tunable, polarization-selective optical switching via anisotropic exciton dynamics in quasi-1D ZrSe_3_

**DOI:** 10.1038/s41377-024-01585-0

**Published:** 2024-09-06

**Authors:** Sang Ho Suk, Sanghee Nah, Muhammad Sajjad, Sung Bok Seo, Jianxiang Chen, Sangwan Sim

**Affiliations:** 1 School of Electrical Engineering, Hanyang University, Ansan, South Korea; 2 Seoul Center, Korea Basic Science Institute, Seoul, South Korea; 3 Nottingham Ningbo China Beacons of Excellence Research and Innovation Institute, University of Nottingham, Ningbo, China; 4 Key Laboratory of Carbonaceous Wastes Processing and Process Intensification Research of Zhejiang Province, University of Nottingham Ningbo China, Ningbo, China

**Keywords:** Ultrafast photonics, Ultrafast photonics, Nanophotonics and plasmonics

## Abstract

In cutting-edge optical technologies, polarization is a key for encoding and transmitting vast information, highlighting the importance of selectively switching and modulating polarized light. Recently, anisotropic two-dimensional materials have emerged for ultrafast switching of polarization-multiplexed optical signals, but face challenges with low polarization ratios and limited spectral ranges. Here, we apply strain to quasi-one-dimensional layered ZrSe_3_ to enhance polarization selectivity and tune operational energies in ultrafast all-optical switching. Initially, transient absorption on unstrained ZrSe_3_ reveals a sub-picosecond switching response in polarization along a specific crystal axis, attributed to shifting-recovery dynamics of an anisotropic exciton. However, its polarization selectivity is weakened by a slow non-excitonic response in the perpendicular polarization. To overcome this limitation, we apply strain to ZrSe_3_ by bending its flexible substrate. The compressive strain spectrally decouples the excitonic and non-excitonic components, doubling the polarization selectivity of the sub-picosecond switching and tripling it compared to that in the tensile-strained ZrSe_3_. It also effectively tunes the switching energy at a shift rate of ~93 meV %^-1^. This strain-tunable switching is repeatable, reversible, and robustly maintains the sub-picosecond operation. First-principles calculations reveal that the strain control is enabled by momentum- and band-dependent modulations of the electronic band structure, causing opposite shifts in the excitonic and non-excitonic transitions. Our findings offer a novel approach for high-performance, wavelength-tunable, polarization-selective ultrafast optical switching.

## Abstract (web summary)

Strain control of quasi-1D ZrSe_3_ enables ultrafast, polarization-selective optical switching with enhanced selectivity and tunable switching energies.

## Graphical abstract

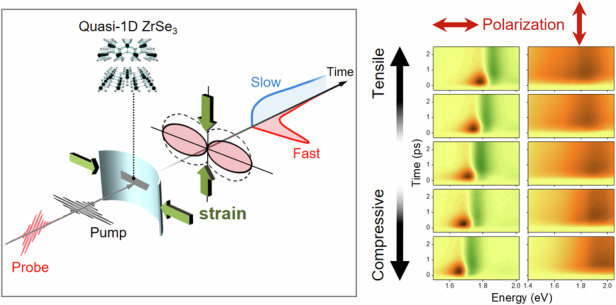

## Introduction

Polarization, which determines the orientation of light wave vibrations, is fundamental to cutting-edge optical technologies used for encoding, transmitting, and storing information across various applications^1–5^. These technologies include polarization division multiplexing in optical communications^2^ and intra-chip interconnects^3^, as well as polarization-driven photonic neuromorphics^4^ and quantum computing^5^. They rely on polarization to efficiently manage and transmit large volumes of data. As such, there is an essential demand for technologies capable of selectively switching and modulating specific polarizations—a key component in encoding polarization-based information onto light and processing target states in polarization-multiplexed signals^2–5^. Such operations require high polarization selectivity to ensure that signals with the target polarization are modulated without affecting other polarization signals^6^. Moreover, to accommodate increasing data volumes, operation within sub-picosecond ranges (i.e., frequencies beyond terahertz) is necessary^7^. Additionally, the recent demands of on-chip nanophotonic devices^8^ necessitate the development of advanced nanomaterials that meet these performance conditions.

Anisotropic two-dimensional materials (A2DMs), such as black phosphorus, group-IV monochalcogenides, rhenium dichalcogenides, and transition-metal tricalcogenides (TMTs), are layered nanomaterials marked by significant directional dependencies in physical characteristics due to their reduced in-plane crystal symmetry^9–11^. Recent focus has been on A2DMs as pivotal for polarization-based nanophotonics, owing to their inherent polarization sensitivity in interactions with light^9–12^. These characteristics of A2DMs, along with their rapid response, strong excitonic effects, and suitability for nanodevice integration, have stimulated extensive research into polarization-dependent sensors and emitters^9–12^. Particularly, A2DMs have shown potential for polarization-selective ultrafast all-optical switches and modulators^13–20^. Challenges remain, however, including non-tunable operational wavelength ranges constrained by the materials’ excitonic or interband transition resonances, and limited polarization selectivity, leading to degraded performance^6,11^.

**Fig. 1:**
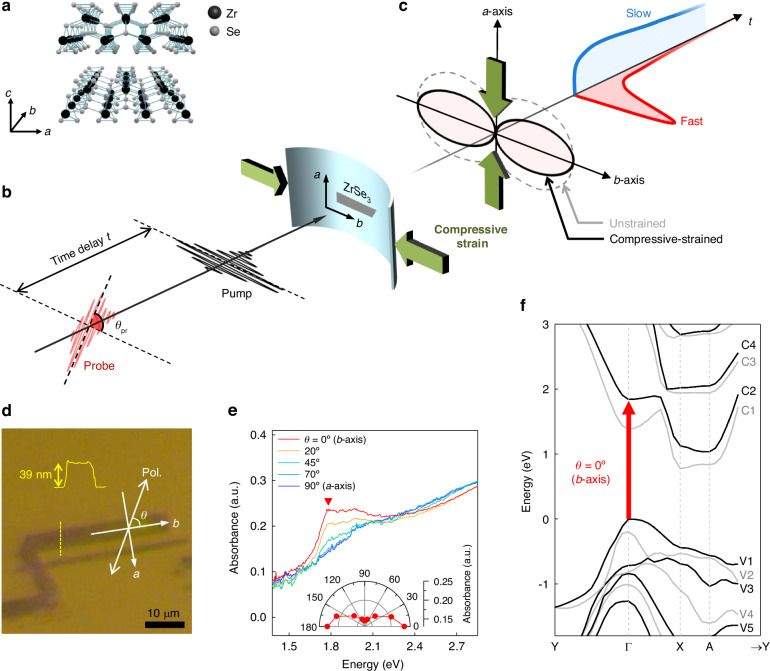
Schematic of strain-tunable ultrafast switching and steady-state characterization. **a** ${\rm{Zr}}{{\rm{Se}}}_{3}$ atomic structure. **b** Schematic of TA experiment. Strain is applied to the ${\rm{Zr}}{{\rm{Se}}}_{3}$ by bending the flexible substrate (green arrows). **c** Illustration of polarization-dependent optical switching: Fast sub-picosecond response along the $b$-axis (red line) while slower response along the $a$-axis (blue line). The dumbbell-shaped dashed and solid polar plots illustrate the polarization-dependent modulation patterns without and with compressive strain, respectively. The green arrows highlight that the compressive strain signifies the modulation’s anisotropy, enhancing the polarization selectivity of the sub-picosecond switching component. **d** Optical image of ${\rm{Zr}}{{\rm{Se}}}_{3}$ crystal on a sapphire substrate is shown with crystal’s $a$- and $b$-axes and the AFM profile along the yellow dashed line. **e** Polarization ($\theta$)-dependent absorbance. The triangle marks the exciton resonance pronounced at $b$-axis polarization. Inset: the absorbance intensity at the exciton peak against $\theta$. **f** The interband transition responsible for the exciton resonance is indicated by the red arrow in the calculated band structure of ${\rm{Zr}}{{\rm{Se}}}_{3}$

Zirconium triselenide (${\rm{Zr}}{{\rm{Se}}}_{3}$), belonging to the TMT family, is an emerging A2DM with unique anisotropic properties. As illustrated in Fig. 1a, each Zr atom bonds to six Se atoms, forming a trigonal prismatic configuration that creates one-dimensional chains along the $b$-axis^21^. These chains intertwine along the $a$-axis to construct a single layer, with successive layers stacked atop one another along the $c$-axis through weak van der Waals forces. Consequently, exfoliated ${\rm{Zr}}{{\rm{Se}}}_{3}$ layers lie predominantly in the $a-b$ plane, often appearing elongated along the $b$-axis^22^. This quasi-one-dimensional (quasi-1D) configuration imparts highly anisotropic optical properties to ${\rm{Zr}}{{\rm{Se}}}_{3}$^21–25^. Notably, excitons, crucial electron-hole pairs governing the optical behavior of low-dimensional semiconductors^26–31^, display pronounced polarization dependence in ${\rm{Zr}}{{\rm{Se}}}_{3}$. Recent research by Li et al. has revealed that mechanical strain along the $b$-axis effectively modulates the resonance energy of excitons in ${\rm{Zr}}{{\rm{Se}}}_{3}$^22^. Moreover, this material exhibits exceptional nonlinear optical characteristics^32,33^, high photoresponsivity^25^ and remarkable air stability^22^. Despite these outstanding attributes, the ultrafast optical properties of ${\rm{Zr}}{{\rm{Se}}}_{3}$‘s anisotropic excitons, including their polarization-dependent dynamic nature, remain unexplored.

In this study, we investigate the strain-tunable, polarization-dependent ultrafast all-optical switching in exfoliated quasi-1D ${\rm{Zr}}{{\rm{Se}}}_{3}$ layers using transient absorption (TA) microscopy (Fig. 1b). We observe an anisotropic modulation feature with sub-picosecond dynamics along the $b$-axis probe polarization, attributed to the exciton’s transient shift associated with hot carrier dynamics. However, the polarization selectivity of this dynamic response is constrained by another slower modulation peak, occurring predominantly at perpendicular polarization due to non-excitonic excited state absorption. To overcome this limitation, we apply uniaxial strain to ${\rm{Zr}}{{\rm{Se}}}_{3}$ by bending a flexible substrate. Under compressive strain, we find that the anisotropy ratio of the sub-picosecond component is enhanced by approximately two-fold, thereby improving the polarization selectivity of the ultrafast modulation (Fig. 1c). Our first-principles calculations reveal that this enhancement stems from mitigating the spectral overlap between switching peaks pronounced in perpendicular polarizations under compressive strain. Additionally, the switching center energy is effectively tuned through the application of strain in a manner that is both repeatable and reversible. These findings underscore the potential of strain-controlled ${\rm{Zr}}{{\rm{Se}}}_{3}$ as an excellent polarization-based ultrafast optical modulation platform with high selectivity and tunable operational wavelengths. Furthermore, this study offers a novel strain-engineering mechanism for manipulating the optical anisotropy of ultrafast exciton dynamics in low-symmetry 2D materials.

## Results

### Steady-state anisotropic absorption

We have first investigated the polarization-dependent steady-state optical properties of unstrained ${\rm{Zr}}{{\rm{Se}}}_{3}$. For this purpose, multilayer ${\rm{Zr}}{{\rm{Se}}}_{3}$ flakes were mechanically exfoliated onto a non-flexible sapphire substrate. An optical image and an atomic force microscopy (AFM) profile of the sample, showing a 39 nm thickness, are presented in Fig. 1d. The flakes extend along the $b$-axis, with the $a$-axis positioned perpendicularly within the crystal plane. To confirm the crystal orientation, we have performed polarization-dependent absorption experiments (Materials and Methods). The light was directed perpendicularly to the flake’s surface, with the polarization angle ($\theta$) relative to the $b$-axis, as indicated in Fig. 1d. The resulting absorbance spectra in Fig. 1e exhibit a pronounced peak at 1.78 eV when polarization is aligned with the $b$-axis ($\theta =0^{\circ}$), accompanied by a high-energy side shoulder. This feature fades as polarization rotates towards the $a$-axis. The inset in Fig. 1e outlines the absorbance intensity at the peak energy across $\theta$, highlighting its linear dichroism, aligning with prior research^22–24^.

This anisotropic resonance is linked to an exciton prominent under $b$-axis polarization^22–24,34,35^. To probe this exciton’s origin, we have investigated the electronic structure and optical transitions through first-principles calculations (see Supplementary Note 1 for calculation details). Fig. 1f shows the band structure of bulk ${\rm{Zr}}{{\rm{Se}}}_{3}$ as derived using the Heyd-Scuseria-Ernzerhof (HSE06) screened hybrid functional. The conduction band minimum (CBM) and valence band maximum (VBM) are located at the X and $\Gamma$ points, respectively, yielding an indirect band gap of ~770 meV, in line with prior studies^22,36^. We designated the conduction bands from bottom to top as C1, C2, … and the valence bands from top to bottom as V1, V2, … For interband transitions between these bands, we have computed the polarization- and momentum-dependent transition dipole moments (TDMs) (Fig. S1). We found that the TDM for the V1 to C2 transition at the Γ point, marked by the red arrow in Fig. 1f, shows significant anisotropy under $b$-axis polarization, exceeding other transitions in intensity. Moreover, the calculated transition energy of 1.84 eV closely aligns with the experimental exciton position at ~1.78 eV. Thus, we associate the Γ-point ${\rm{V}}1\to {\rm{C}}2$ transition with the interband absorption related to the exciton. The ~60 meV difference between the calculated transition and the observed exciton position probably indicates the exciton binding energy, given the ~49 meV value for the bulk ${\rm{Zr}}{{\rm{Se}}}_{3}$^24^.

### Anisotropic polarization-dependent ultrafast modulation

Based on the understanding of steady-state optical characteristics, we have investigated ultrafast dynamics in ${\rm{Zr}}{{\rm{Se}}}_{3}$ using TA microscopy. As depicted in Fig. 1b, the pump pulse excites the ${\rm{Zr}}{{\rm{Se}}}_{3}$ flake, causing a change in the intensity of the probe pulse that transmits through the sample. This change is expressed as $\Delta T={T}_{{\rm{pump}}}-{T}_{0}$, where ${T}_{{\rm{pump}}}$ (${T}_{0}$) denotes the probe intensity with (without) the pump. We have recorded $\Delta T/{T}_{0}$ as a function of the pump-probe time delay ($t$) and probe energy (see “Materials and methods” section for details).

**Fig. 2:**
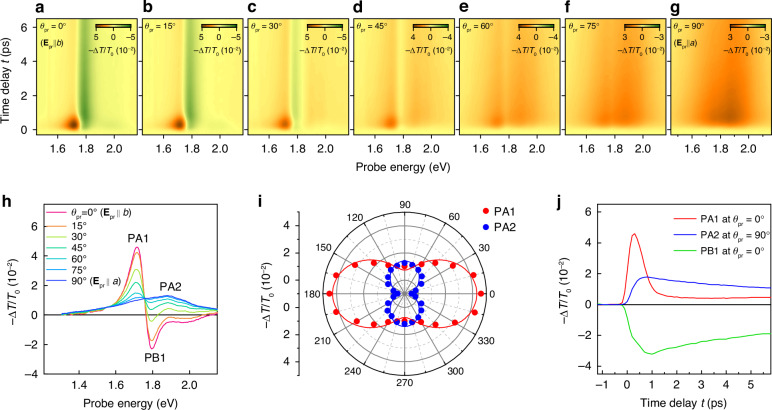
Polarization-dependent ultrafast dynamics. **a**–**g** TA maps measured at various probe polarization angles (${\theta }_{{\rm{pr}}}$) relative to the $b$-axis, with pump polarization fixed along the $b$-axis. **h** Corresponding ${\theta }_{{\rm{pr}}}$-dependent line-cut TA spectra at $t=0.3$ ps, highlighting peaks pronounced at the $b$-axis polarization (PA1 and PB1) and at the $a$-axis polarization (PA2). **i** $-\Delta T/{T}_{0}$ intensities of PA1 and PA2 versus ${\theta }_{{\rm{pr}}}$. Solid lines are cosine squared fits. **j** TA traces at peaks PA1, PA2, and PB1

Before exploring strain control, we first performed TA experiments on unstrained ${\rm{Zr}}{{\rm{Se}}}_{3}$ on a rigid sapphire substrate (Fig. 1d), aiming to focus on the polarization-dependent dynamics. Utilizing a 3.1 eV pump with polarization along the $b$-axis, we measured TA dynamics while varying the probe polarization angle (${\theta }_{{\rm{pr}}}$) relative to the $b$-axis. The pump fluence was set as 30 μJ ${{\rm{cm}}}^{-2}$. Resulting TA maps in Fig. 2a–g exhibit significant polarization dependence on both spectral and temporal aspects. To examine the polarization spectral changes in detail, we plot line-cut $-\Delta T/{T}_{0}$ profiles at a fixed time delay ($t=0.3$ ps) in Fig. 2h. At ${\theta }_{{\rm{pr}}}=0^{\circ}$, pronounced photo-bleaching (i.e., $-\Delta T/{T}_{0} < 0$) is observed above ~1.76 eV. We designate a sharp photo-bleaching peak at 1.79 eV as PB1, whose position closely matches the exciton resonance depicted in Fig. 1e. Similarly, PB1 exhibits a shoulder on its high-energy side and a maximal response when polarized along the $b$-axis, mirroring the characteristics of the exciton’s absorption spectrum. This suggests that PB1 results from the bleaching of the exciton.

Photo-induced absorption (i.e., $-\Delta T/{T}_{0} > 0$) peaks are observed at 1.72 eV and 1.88 eV, labeled as PA1 and PA2, respectively (Fig. 2h). They exhibit several contrasting features. First, while PA1 displays a sharp profile, PA2 appears relatively broad with lower amplitude. Second, PA1 is pronounced at ${\theta }_{{\rm{pr}}}=0^{\circ}$, whereas PA2 is maximized at ${\theta }_{{\rm{pr}}}=90^{\circ}$. This orthogonal polarization dependence is also evident in Fig. 2i, where ${\theta }_{{\rm{pr}}}$-dependent $-\Delta T/{T}_{0}$ values at each peak energy are displayed. Third, PA1 and PA2 exhibit different dynamics, as shown in Fig. 2j. PA1 exhibits a rapid rise, followed by a sub-picosecond decay with a time constant of ~0.4 ps. In contrast, PA2 slowly reaches its maximum at $t$ $\approx 0.8$ ps and shows decay dynamics significantly slower than that of PA1.

Among the peaks discussed, namely PB1, PA1, and PA2, PA1 is particularly notable. It demonstrates the highest amplitude, fastest sub-picosecond response, and polarization-dependent on/off feature, making it promising for THz range polarization-selective ultrafast switching. We later aim to enhance its polarization selectivity and tune the operational wavelength through strain application.

Additionally, $-\Delta T/{T}_{0}$ profiles exhibit no significant dependence on pump polarization (Fig. S2). This result can be interpreted as the loss of initial polarization memory due to rapid momentum scattering of pump-excited carriers^11,19^.

### Origin of polarization-dependent dynamics

**Fig. 3:**
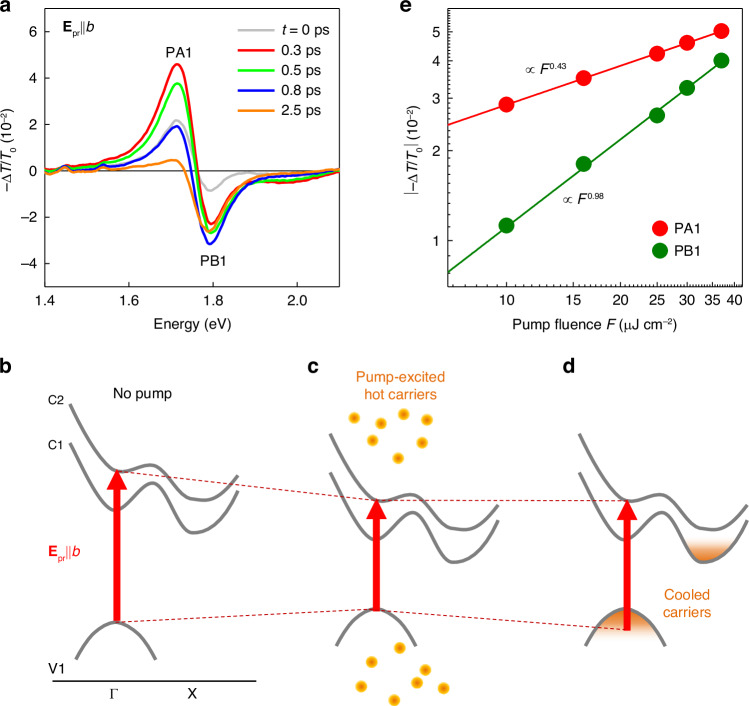
**Exciton dynamics at**${\boldsymbol{b}}$**-axis probe polarization**. **a** TA spectra at several pump-probe time delays, extracted from Fig. 2a. Peak positions of PA1 and PB1 are marked. **b**–**d** Schematics of pump-induced ultrafast dynamics. Gray lines represent the simplified band structure of ${\rm{Zr}}{{\rm{Se}}}_{3}$. The red arrows indicate the interband transition for the exciton pronounced at ${{\bf{E}}}_{{\rm{pr}}}{\rm{||}}b$ without pump excitation (**b**), in the hot carrier regime immediately after the pump (**c**), and after carriers cool down (**d**). Changes in the arrow length are associated with dynamic shifts in exciton energy. **e** Pump fluence-dependent absolute TA intensities for PA1 and PB1 (dots) alongside power-law fits (lines)

The observed TA peaks—PA1, PA2, and PB1—were pronounced when the probe polarization was parallel to either the $b$-axis (${{\bf{E}}}_{{\rm{pr}}}{||b}$) or the $a$-axis (${{\bf{E}}}_{{\rm{pr}}}{||a}$). Thus, we have discussed detailed dynamics at these two significant polarizations, respectively. Let us examine the ${{\bf{E}}}_{{\rm{pr}}}{\rm{||}}b$ polarization first. Fig. 3a depicts $-\Delta T/{T}_{0}$ profiles at ${{\bf{E}}}_{{\rm{pr}}}{\rm{||}}b$ across several time points, taken from Fig. 2a. At $t=0.3$ ps, PA1 reaches its maximum, with its line-shape mirroring the derivative form of the absorption spectrum, suggesting that $-\Delta T/{T}_{0}$ is predominantly influenced by the exciton redshift^37^ (Fig. S3). Subsequently, PA1 quickly diminishes as a result of the exciton redshift’s mitigation, whereas PB1 reaches its peak intensity at $t=0.8$ ps, indicating the dominance of exciton bleaching. These assignments are supported by the further fitting analysis of the temporal evolution of the $-\Delta T/{T}_{0}$ profile (Fig. S4).

Such dynamics can be understood through the processes depicted in Fig. 3b–d. The red arrow in Fig. 3b denotes the ${\rm{V}}1\to {\rm{C}}2$ transition at the $\Gamma$ point in the absence of the pump. The exciton energy corresponds to the energy difference between this transition and the exciton binding energy. Immediately after pump excitation, the generated hot charge carriers screen the repulsive Coulomb interaction, causing a shrinkage of the bandgap. This phenomenon, known as bandgap renormalization (BGR)^37–41^, reduces the ${\rm{V}}1\longrightarrow {\rm{C}}2$ transition energy, as illustrated by the shortened arrow in Fig. 3c, leading to exciton redshift. The pump-generated carriers can also lead to blueshift in exciton by reducing the exciton binding energy via screening of attractive Coulomb interaction between electrons and holes^38^. Therefore, the direction of the exciton shift is determined by the competition between these two opposing effects. In our experiment, the exciton redshift was clearly observed, indicating the dominance of BGR. The hot carriers then relax to the band edge via carrier-phonon scattering. Consequently, most electrons occupy states near the CBM at the X point, while holes accumulate near the VBM at the $\Gamma$ point (Fig. 3d). The latter process leads to an effective increase in the energy of the $\Gamma$-point ${\rm{V}}1\longrightarrow {\rm{C}}2$ transition (Burstein-Moss effect)^42^, as illustrated by the re-lengthened arrow in Fig. 3d. This counteracts the exciton redshift due to BGR, explaining the rapid decay of PA1. Additionally, the filling of VBM states by cooled holes leads to exciton bleaching (i.e., PB1). This elucidates why PB1 grows alongside the decay of PA1 (Fig. 3a). We further validated this scenario through quantitative estimation of BGR, exciton binding energy, and Burstein-Moss shift (see Supplementary Note 2 for details).

To further confirm our interpretation, we have performed pump fluence ($F$)-dependent TA measurements. The maximum intensity of PB1 exhibits a nearly linear $F$ dependence (Fig. 3e). This aligns with the fact that exciton bleaching scales linearly with the excited carrier density^43^. In contrast, PA1 follows a power law of ${F}^{0.43}$, agreeing with the sublinear carrier density-dependent characteristic of BGR^37^. We also observed that both the decay of PA1 and the rise of PB1 are delayed as $F$ increases (Fig. S5), which agrees with the typical characteristics of carrier cooling dynamics^44^ in our scenario.

Next, we have investigated the origin of the PA2 pronounced in ${{\bf{E}}}_{{\rm{pr}}}{||a}$ polarization. As shown in Fig. 2j, the PA2 trace exhibits markedly different dynamics from the PA1 dynamics but resembles that of opposite-signed PB1. This suggests that PA2, like PB1, arises from carrier populations relaxed to the band edge. Carriers occupying the band edge can induce photo-induced absorption by absorbing probe photons and transitioning to higher energy states. Hence, this mechanism, known as excited state absorption (ESA)^18,45^, emerges as a highly plausible explanation for PA2. This possibility is supported by the linear $F$ dependence of the maximum intensity of PA2 (Fig. S6a), given that ESA scales proportionally with the pump-excited carrier density^18^.

**Fig. 4:**
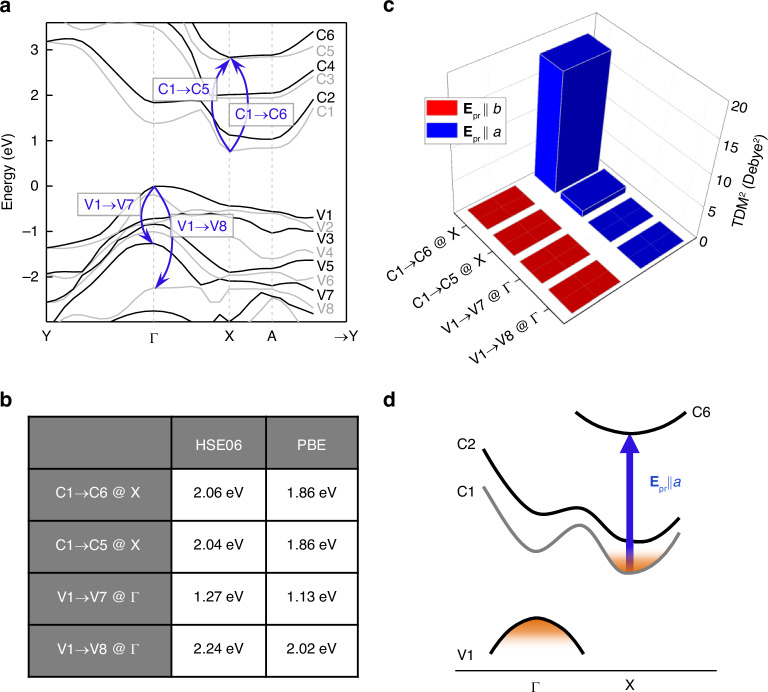
**Origin of PA2 at**${\boldsymbol{a}}$**-axis probe polarization**. **a** Arrows in the ${\rm{Zr}}{{\rm{Se}}}_{3}$ band structure represent four possible ESA transitions considered to explain PA2. **b**, **c** Energies for these transitions calculated using HSE06 and PBE methods (**b**), and their probe polarization-resolved transition dipole moments (**c**). **d** Among the four transition candidates, our analysis attributes the X-point ${\rm{C}}1\to {\rm{C}}6$ transition as PA2, as represented by the blue arrow in the simplified band structure

We have further theoretically verified this possibility. Fig. 4a illustrates the HSE06 electronic structure of ${\rm{Zr}}{{\rm{Se}}}_{3}$, where it can be assumed that most of the relaxed electrons and holes reside predominantly at the CBM of the X point and the VBM of the $\Gamma$ point, respectively. Among possible ESA transitions involving these CBM and VBM states, we have selected four transitions whose energies fall within or near our spectral window, as indicated by the arrows in Fig. 4a. Their transition energies obtained from the HSE06 band structure, along with those from the Perdew-Burke-Ernzerhof (PBE) functional (Fig. S7), are presented in Fig. 4b. We have computed the polarization-resolved transition dipole moments (TDMs) for these transitions (Fig. 4c). Here, the TDM of the C1→C6 transition at the X point exhibits the highest value at ${{\bf{E}}}_{{\rm{pr}}}{||a}$ compared to others, while it nearly vanishes at ${{\bf{E}}}_{{\rm{pr}}}{||b}$. This behavior agrees with the observed polarization dependence of PA2 in Fig. 2h. Furthermore, the energy of the C1 → C6 transition (1.86 eV for PBE, 2.06 eV for HSE06) is consistent with the peak position of PA2 (1.88 eV). These results suggest that PA2 is predominantly attributed to the C1 → C6 ESA of electrons at the X point, as illustrated in Fig. 4d. This ESA is not a response involving electron-hole pairs, but rather a transition of electrons alone, which indicates the non-excitonic nature of PA2. Based on this assignment, we additionally investigated the decay dynamics of PA2. It exhibits bi-exponential decay with time constants of ~3-4 ps and ~30-50 ps, which we attributed to Auger recombination and defect trapping, respectively (Fig. S6).

### Strain-engineering of polarization-driven optical switching

The TA dynamics explored thus far provide crucial insights into ultrafast optical switching using ${\rm{Zr}}{{\rm{Se}}}_{3}$. The scheme of all-optical switching employs the pump pulse as the control light and the probe pulse, modulated by it, as the signal carrier^7,11^. Thus, effective modulation hinges on significant TA peaks, namely PA1, PB1, and PA2, with PA1 being the most pronounced and spectrally defined. Also, the sub-picosecond response of PA1 makes it suitable for high-frequency applications in the THz range. Moreover, PA1 shows marked probe polarization sensitivity in the $b$-axis polarization, enabling the selective switching of polarization-multiplexed signals.

Nonetheless, limitations exist. The spectral position of PA1 is fixed by exciton resonance, restricting switching wavelength flexibility. More importantly, the anisotropy ratio is also limited. As depicted in Fig. 2h, while the PA1 is maximized at ${{\bf{E}}}_{{\rm{pr}}}{\rm{||}}b$, broad PA2 grows as the polarization rotates to the orthogonal direction, retaining a residual modulation amplitude even at ${{\bf{E}}}_{{\rm{pr}}}{\rm{||}}a$. Such incomplete anisotropy can lead to unwanted partial switching of non-targeted polarization components, resulting in signal distortion. Quantitatively, the anisotropy ratio of the switching at PA1’s peak is

1 $$\rho =\frac{{\left[-\Delta T/{T}_{0}\right]}_{b-{\rm{pol}}.}}{{\left[-\Delta T/{T}_{0}\right]}_{a-{\rm{pol}}.}}\approx 4.6$$

where ${\left[-\Delta T/{T}_{0}\right]}_{b-{\rm{pol}}.}$ and ${\left[-\Delta T/{T}_{0}\right]}_{a-{\rm{pol}}.}$ denote the TA signals at ${{\bf{E}}}_{{\rm{pr}}}{||b}$ and ${{\bf{E}}}_{{\rm{pr}}}{||a}$, respectively. Although this ratio is comparable to those in other A2DMs that show sub-picosecond anisotropic responses^13,46^, enhancing it is imperative for developing high-performance devices.

**Fig. 5:**
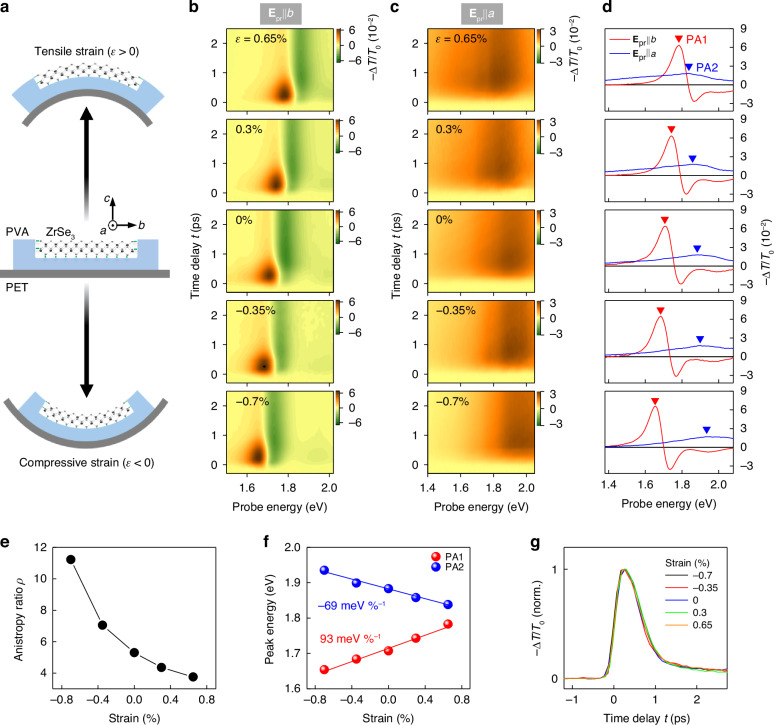
Strain-controlled polarization-dependent all-optical switching. **a** Schematic illustration of strain application method. ${\rm{Zr}}{{\rm{Se}}}_{3}$ coated with PVA is bonded to PET substrate (middle), which is then bent to introduce uniaxial tensile (top) or compressive strain (bottom) along the $b$-axis. **b**, **c** TA maps measured under different strains with probe polarization parallel to the $b$-axis (**b**) and the $a$-axis (**c**). **d** Corresponding TA profiles at $t=0.3$ ps for both polarizations are shown with triangles marking the peak positions of PA1 and PA2. **e** Anisotropy ratio of polarization-dependent switching at the peak position of PA1 is presented as a function of strain. **f** Strain-dependent peak positions of PA1 and PA2 (dots), and their linear fits (lines) with shift ratios. **g** Normalized TA traces at PA1 under different strains

To overcome these limitations, we have utilized mechanical strain on ${\rm{Zr}}{{\rm{Se}}}_{3}$. Fig. 5a illustrates the strain induction method: a ${\rm{Zr}}{{\rm{Se}}}_{3}$ crystal flake coated with polyvinyl alcohol (PVA) is affixed to a flexible polyethylene terephthalate (PET) substrate^47^, then strain ($\varepsilon$) was introduced by bending the substrate (see Fig. S8 for details). Upward bending applies tensile strain ($\varepsilon \,>\, 0$) to the crystal, while downward bending results in compressive strain ($\varepsilon \,<\, 0$)^48^. By varying the bending degree, we controlled the strain, calculated from the substrate’s thickness and curvature radius (Fig. S8). The PVA coating ensures that flakes remain securely on the substrate, preventing slipping^47^. Notably, Li et al. recently demonstrated^22^ that $b$-axis-directed strain more effectively shifts excitons in ${\rm{Zr}}{{\rm{Se}}}_{3}$ than $a$-axis strain. Motivated by this discovery, we conducted ultrafast TA experiments while applying uniaxial strain along the $b$-axis (Fig. S8).

Fig. 5b, c present the measured strain-dependent TA dynamics at ${{\bf{E}}}_{{\rm{pr}}}{\rm{||}}b$ and ${{\bf{E}}}_{{\rm{pr}}}{\rm{||}}a$ polarizations, respectively, under 3.1 eV, 50 μJ ${{\rm{cm}}}^{-2}$ pump with $b$-axis polarization. Fig. 5d displays the corresponding $-\Delta T/{T}_{0}$ profiles at a fixed time ($t=0.3$ ps), marking the peak positions of PA1 and PA2 with triangles. As strain transitions from tensile to compressive, PA1 at ${{\bf{E}}}_{{\rm{pr}}}{\rm{||}}b$ redshifts, but PA2 at ${{\bf{E}}}_{{\rm{pr}}}{\rm{||}}a$ blueshifts. These opposite shift trends mitigate the spectral overlap between PA1 and PA2, enhancing the polarization selectivity of optical switching at PA1. Fig. 5e shows the strain-dependent anisotropy ratio $\rho$ (Eq. (1)) at the PA1 peak, where the maximum compressive strain ($\varepsilon =-0.7 \%$) exhibits an anisotropy ratio of $\rho \approx 11.2$, approximately 2 times greater than that under the unstrained condition, and ~3 times larger than that under maximum tensile strain ($\varepsilon =0.65 \%$). These results underline that strain application boosts polarization selectivity of ultrafast all-optical switching by shifting PA1 and PA2 in opposite directions. It is also worth mentioning that, in addition to spectral shifts, strain induces slight line-shape variations in PA2, which may partially affect the anisotropy ratio enhancement (Fig. S9).

Furthermore, the observed strain dependence of TA underscores the potential for precise switching wavelength tuning. Fig. 5f depicts the strain-induced shifts of PA1 and PA2 and their linear fits, highlighting a notable shift rate for PA1 of $93$ ${\rm{meV}}$ ${ \% }^{-1}$. This rate is consistent with those observed in previous steady-state reflection measurements of ${\rm{Zr}}{{\rm{Se}}}_{3}$^22^ and surpasses the shift rates found in typical transition metal dichalcogenides by ~1.5-3 times^49^. Our experiments confirm the repeatability of the peak shift through multiple strain-relaxation cycles (Fig. S10), and the high stability of the strain tunability after 20 days of air exposure (Fig. S11). The total tuning range of the PA1 peak energy (wavelength) over the entire range of applied strains is 1.65–1.78 eV (~697–751 nm). The strain-dependent shift rate for the exciton bleaching position (PB1) mirrors that of PA1, reinforcing the evidence of exciton shifting (Fig. S12). The PA2’s strain-dependent shift rate is $-69$ ${\rm{meV}}$ ${ \% }^{-1}$ (blue dots in Fig. 5f), with its magnitude smaller than that of PA1. This result is consistent with the theoretical analysis to be discussed later.

We also note that PA1’s sub-picosecond response remains stable under strain (Fig. 5g), indicating that band structure alterations weakly impact hot carrier cooling dynamics. After the initial fast dynamics, a slow component due to the lasting carrier population is observed, which could possibly be suppressed through various strategies such as defect engineering^50^ and destructive interference^51^. Additionally, PA2’s longer-time-scale dynamics show minimal strain sensitivity (Fig. S13). Thus, strain application in ${\rm{Zr}}{{\rm{Se}}}_{3}$‘s all-optical switching ensures consistent temporal responses while enhancing polarization selectivity and facilitating operational wavelength tuning.

We have determined that the maximum allowable compressive strain is -0.7%, and the corresponding limit of the anisotropy ratio ranges from ~10 to 11 (Fig. S14). This limitation is probably due to the delamination of the flake from the substrate caused by buckling under compressive strain^52,53^.

### Theoretical analysis of strain tuning

**Fig. 6:**
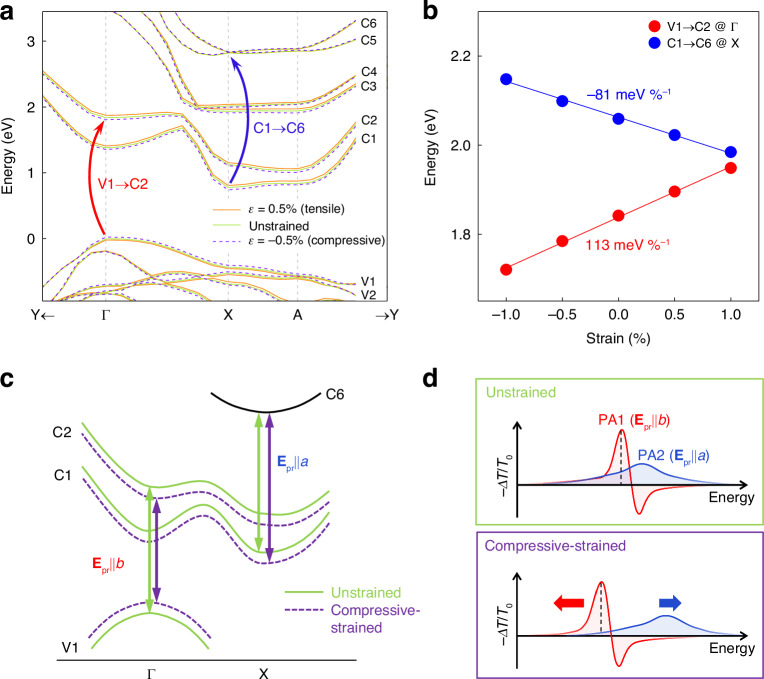
**Theoretical analysis of strain tuning**. **a** Strain-dependent band structures of ${\rm{Zr}}{{\rm{Se}}}_{3}$ under $b$-axis uniaxial strain. The red arrow represents the exciton-related ${\rm{V}}1\to {\rm{C}}2$ transition pronounced in $b$-axis polarization, while the blue arrow represents ${\rm{C}}1\to {\rm{C}}6$ transition responsible for ESA in $a$-axis polarization. **b** Energies of these two transitions against strain (dots), with their linear fits (lines) and shift ratios. **c**, **d** Schematics depicting band structure changes due to compressive strain (**c**) and the resulting TA profile changes (**d**). Strain decreases the energy of the $\Gamma -$ point ${\rm{V}}1\to {\rm{C}}2$ transition (**c**), causing PA1’s redshift for $b$-axis polarization (red curved in **d**). In contrast, strain increases the X-point ${\rm{C}}1\to {\rm{C}}6$ transition energy (**c**), resulting in PA2’s blueshift for $a$-axis polarization (blue curves in **d**). Their opposite shifting enhances the anisotropy ratio at the PA1 peak, marked by dashed black lines in **d**

To confirm the modulation peak shifts, we have analyzed the strain-dependent band structure. Fig. 6a shows the HSE06 structures of bulk ${\rm{Zr}}{{\rm{Se}}}_{3}$ under both 0.5% tensile and compressive uniaxial strains along the b-axis, alongside their unstrained counterpart. Applying compressive strain causes C2 to descend while V1 ascends. As a result, the ${\rm{V}}1\to {\rm{C}}2$ transition energy at Γ decreases, aligning with the redshift of PA1 observed under compressive strain in Fig. 5d. On the other hand, under compressive strain, while C1 descends, C6 stays relatively stable at the X point, leading to a rise in the energy of the X-point ${\rm{C}}1\to {\rm{C}}6$ transition, mirroring the compressive strain-induced blueshift of PA2 (Fig. 5d).

Fig. 6b shows the calculated energies for the $\Gamma$-point ${\rm{V}}1\to {\rm{C}}2$ and X-point ${\rm{C}}1\to {\rm{C}}6$ transitions versus strain, with shift rates of 113 ${\rm{meV}}$ ${ \% }^{-1}$ and $-81$ ${\rm{meV}}$ ${ \% }^{-1}$ respectively. These rates are slightly higher than those observed experimentally in Fig. 5f, possibly due to incomplete transmission of applied strain to the flake in experiments. However, the ratio of their absolute shift rates, approximately $\frac{113{\rm{meV}}{ \% }^{-1}}{81{\rm{meV}}{ \% }^{-1}}\approx 1.40$, aligns well with the experimental ratio of $\frac{93{\rm{meV}}{ \% }^{-1}}{69{\rm{meV}}{ \% }^{-1}}\approx 1.35$ for PA1 and PA2 in Fig. 5f. This result supports our observation that PA2 is less sensitive to strain than PA1. According to previous research^22^, the C2 state at Γ is influenced by the Zr ${d}_{{xy}}$ orbital, and the C6 state at X by the Zr ${d}_{{xz}}$ orbital. Therefore, strain along the $b$-axis ($y$-direction) more significantly impacts the former, partially explaining the greater sensitivity of PA1 to strain than PA2. However, this rationale doesn’t extend to the V1 at Γ and C1 at X states, primarily affected by Se ${p}_{z}$ and Se ${p}_{x}$ orbitals, respectively^22^, necessitating further research to fully understand strain-dependent band shifts.

Fig. 6c, d summarizes the effect of compressive strain on enhancing polarization selectivity in optical switching. Under compressive strain, the energy of the ${\rm{V}}1\to {\rm{C}}2$ transition at the $\Gamma$ point decreases, while that of the C1 → C6 transition at the X point increases (Fig. 6c). The former change leads to the redshift for the exciton-originated PA1 at ${{\bf{E}}}_{{\rm{pr}}}{\rm{||}}b$ polarization, while the latter results in the blueshift for the non-excitonic ESA-driven PA2 at ${{\bf{E}}}_{{\rm{pr}}}{\rm{||}}a$ (Fig. 6d), thereby increasing the modulation anisotropy ratio at the PA1 peak (indicated by dashed lines in Fig. 6d).

### Comparison with other polarization-dependent all-optical switching studies

**Fig. 7:**
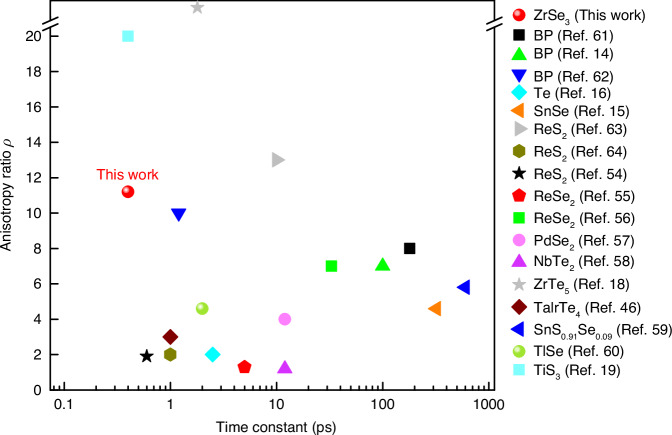
**Comparison of switching parameters**. This figure compares the maximum anisotropy ratio and time constant obtained in this study with those obtained from polarization-dependent optical pump-probe studies on other A2DMs. The result from ref. 18 (gray star) reports an infinitely high anisotropy ratio

We have compared the switching performance obtained from ${{\rm{ZrSe}}}_{3}$ with those of other materials and systems. Fig. 7 compares the two key parameters: maximum anisotropic ratios and the shortest response time constants obtained from polarization-dependent ultrafast optical experiments on various A2DMs^14–16,18,19,46,54–64^. We can see that ${{\rm{ZrSe}}}_{3}$ exhibits a faster response time and a higher anisotropic ratio compared to most other materials. Some other materials, such as BP^62^, ${{\rm{ZrTe}}}_{5}$^18^, and ${{\rm{TiS}}}_{3}$^19^, show short response time constants around 1 ps and have anisotropy ratios similar to or higher than ${{\rm{ZrSe}}}_{3}$. However, BP and ${{\rm{ZrTe}}}_{5}$ have poor air stability^65,66^, while ${{\rm{ZrSe}}}_{3}$ demonstrates excellent stability in ambient conditions^22^ (Fig. S11). Moreover, the studies on BP and ${{\rm{ZrTe}}}_{5}$ did not present the tunability of the switching wavelength. ${{\rm{TiS}}}_{3}$ is known to have relatively high air stability compared to these materials^19^. However, the modulation depth in ${{\rm{TiS}}}_{3}$ is an order of magnitude lower than that of ${{\rm{ZrSe}}}_{3}$. Moreover, the tunability of the switching wavelength in ${{\rm{TiS}}}_{3}$ has not been investigated, thus it is still limited to a narrow near-infrared peak. We expect that the experimental methods provided in our study can be applied to ${{\rm{TiS}}}_{3}$ to tune the operation wavelength and further enhance the anisotropy ratio, as it was recently predicted that the optical resonance and anisotropy of this material can be controlled by compressive strain^67^.

Beyond A2DMs, there has been significant research on polarization-dependent ultrafast optical switching based on metamaterials and plasmonic structures, with sub-picosecond switching speeds and high anisotropy ratios^7,68,69^. However, such systems require specific patterns and structures to achieve polarization selectivities and target optical resonances, resulting in fixed devices that lack reversible tunability. Moreover, relatively complex fabrication processes such as patterning and integration of multiple components are often required. In contrast, A2DMs inherently possess optical anisotropy, eliminating the need for complex fabrications. Also, their high flexibility allows for easy modulation of optical resonance through strain. Particularly, ${{\rm{ZrSe}}}_{3}$ is promising for strain-tunable ultrafast nanophotonic technologies due to its relatively high resonance modulation rate under strain among A2DMs.

## Discussion

In conclusion, we have shown that applying strain effectively controls the ultrafast, polarization-driven all-optical switching in ${\rm{Zr}}{{\rm{Se}}}_{3}$. Initially, using polarization-resolved TA microscopy, we identified strongly anisotropic ultrafast dynamics. At the $b$-axis parallel probe polarization, we detected the modulation peak (PA1) with a sub-picosecond response, attributed to exciton redshift due to bandgap renormalization in the hot carrier regime and its subsequent recovery due to carrier cooling. Conversely, at the perpendicular $a$-axis probe polarization, a slower, broader peak (PA2) emerged, linked to the non-excitonic excited state absorption of electrons. From a fundamental perspective, the coexistence of these distinct transients in perpendicular polarizations offers new insights into the ultrafast light-matter interactions in anisotropic systems. However, from an application standpoint, PA1 and PA2 are spectrally overlapped, limiting the polarization selectivity of optical switching. To overcome this, we applied uniaxial strain along the $b$-axis to ${\rm{Zr}}{{\rm{Se}}}_{3}$ by bending the flexible substrate. Compressive strain caused opposite peak shifts in PA1 and PA2, reducing their overlap and doubling the modulation anisotropy ratio compared to the unstrained condition, thereby enhancing polarization selectivity of the sub-picosecond optical switching. Furthermore, applying strain effectively tuned the modulation’s center energy, achieving a shift rate of 93 ${\rm{meV}}{ \% }^{-1}$. This strain engineering method was proven to be repeatable, reversible, and consistently maintained sub-picosecond dynamics.

We emphasize that our discovery introduces a novel concept in strain-engineering photonics. Leveraging the high strain sensitivity and flexibility of 2D materials, recent studies have intensively explored the modulation of their various optical properties through strain^27,30,70,71^. Despite this, prior ultrafast studies on strain-manipulated 2D materials predominantly focused on isotropic substances^72–75^, leaving anisotropic dynamics unexplored. Although some steady-state optical studies explored strain engineering in A2DMs, the emphasis has been largely on the influence of strain direction^22,76^. Contrary to the previous studies, our work pioneers the significant enhancement of anisotropy in ultrafast dynamics through strain. Our methodology, which leverages strain to induce opposing spectral shifts in components under perpendicular polarizations, offers a novel approach to amplify polarization selectivity in ultrafast nonlinear optical responses. This advancement mitigates the modulation of non-targeted polarization components, possibly elevating modulation efficiency for polarization-multiplexed signals. Thus, this work might propel advancements across a wide array of polarization-based technologies, encompassing optical communications, intra-chip interconnects, and neuromorphic photonics^2–5^.

## Methods

### Absorption microscopy

Steady-state absorption measurements were conducted with a commercial microspectrophotometer (20/30 PV, Craic Technologies) at the Next Generation Display Research Core Facility Hanyang University ERICA. To set the light’s polarization angle, a wire-grid polarizer was utilized. The experiment was conducted under ambient conditions.

### Ultrafast TA microscopy

We utilized a Yb:KGW amplifier system (Light Conversion, PHAROS) operating at 100 kHz, delivering pulses of 220 fs duration and an output power of 20 W. The amplifier’s output was split into two paths. One part fueled a collinear optical parametric amplifier system (Light Conversion, ORPHEUS) to produce the pump beam centered at 400 nm. The remainder was directed onto a sapphire crystal to generate broadband probe light. Within the TA microscope module (Light Conversion, Harpia-TA), a beam splitter combined the pump and probe beams, focusing them onto the sample via a 40x objective. The transmitted probe beam was collected by a 20x objective, then spectrally dispersed in a monochromator and captured by a silicon-based array detector. Beam intensities were regulated using neutral density filters. All experiments were conducted under ambient conditions. For the strain-dependent TA experiments, PVA-coated ${\rm{Zr}}{{\rm{Se}}}_{3}$ on PET substrates were mounted on a stage for mechanical bending, within the TA microscope module (for details, see Fig. S8).

## Supplementary information

Supplementary Information for “Sub-picosecond, strain-tunable, polarization-selective optical switching via anisotropic exciton dynamics in quasi-1D ZrSe3”

## Data Availability

The data that support the plots within this paper and other findings of this study are available within this article and its Supplementary Information file, and are also available from the corresponding author upon request.
