# Supplementary material for: Sub-picosecond, strain-tunable, polarization-selective optical switching via anisotropic exciton dynamics in quasi-1D ZrSe3
Source: Light Sci Appl. 2024 Sep 6;13:240. doi: 10.1038/s41377-024-01585-0 (PMC11377565; doi:10.1038/s41377-024-01585-0)
Supplement: Supplementary file 1 — Supplementary Information for “Sub-picosecond, strain-tunable, polarization-selective optical switching via anisotropic exciton dynamics in quasi-1D ZrSe3” [file 41377_2024_1585_MOESM1_ESM.pdf]

Supplementary Information for "Sub-picosecond, strain-tunable, polarization-selective optical switching via anisotropic exciton dynamics in quasi-1D $ZrSe_3$"

Sang Ho Suk[1†], Sanghee Nah[2†], Muhammad Sajjad[3,4†], Sung Bok Seo[1†], Jianxiang Chen[1] and Sangwan Sim[1]*

[1]School of Electrical Engineering, Hanyang University, Ansan, 15588, South Korea
[2]Seoul Center, Korea Basic Science Institute, Seoul 02841, South Korea
[3]Nottingham Ningbo China Beacons of Excellence Research and Innovation Institute, University of Nottingham, Ningbo 315100, China
[4]Key Laboratory of Carbonaceous Wastes Processing and Process Intensification Research of Zhejiang Province, University of Nottingham Ningbo China, Ningbo 315100, China

*Corresponding author: Sangwan Sim (swsim@hanyang.ac.kr)
[†]These authors contributed equally to this work.

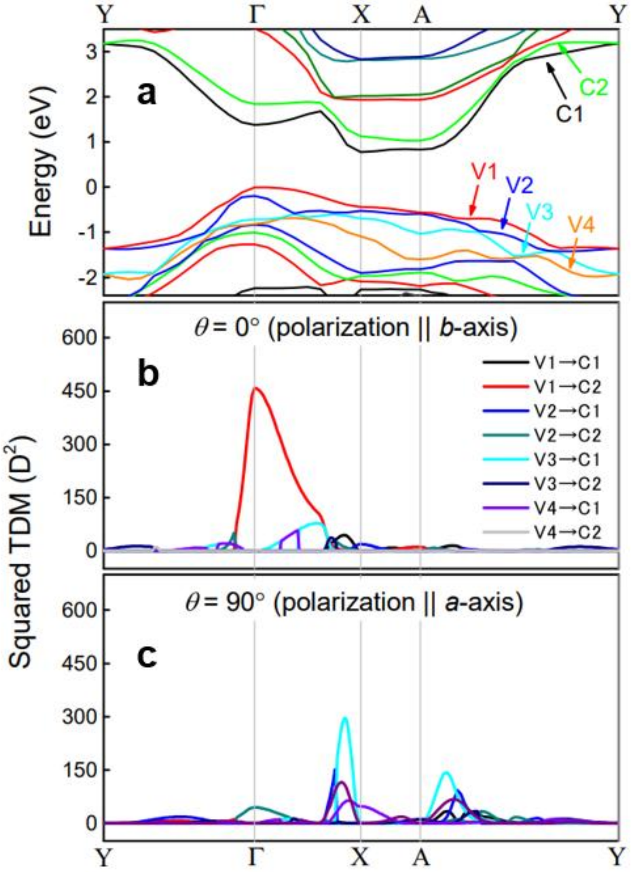

Fig. S1 | Theoretical analysis of the anisotropic exciton resonance.

a The HSE06 band structure of bulk $ZrSe_3$ is depicted with the conduction bands denoted as C1 and C2, and the valence bands labeled as V1, V2, V3, V4. To identify the transition responsible for the observed exciton peak, we examine the interband transitions between these bands.

b,c Panels (**b**) and (**c**) depict the squared transition dipole moments (TDMs) for interband transitions under light polarization parallel to the b-axis ($\theta = 0°$) and parallel to the a-axis ($\theta = 90°$), respectively. The $V1 \rightarrow C2$ transition at the Γ point exhibits a significant TDM in b-axis polarization (red line in (**b**)), while it diminishes considerably in a-axis polarization (**c**). This observation aligns with the anisotropy of the exciton peak in Fig. 1e of the main text. Furthermore, the energy of the $V1 \rightarrow C2$ transition at Γ is 1.84 eV in the band structure in (**a**), which roughly matches the exciton peak energy of 1.78 eV. The energy difference of 60 meV closely resembles the experimentally estimated exciton binding energy of 49 meV in bulk $ZrSe_3$[1]. Therefore, we attribute the $V1 \rightarrow C2$ transition at the Γ point as the interband transition responsible for the observed exciton, as indicated by the red arrow in Fig. 1f of the

main text.

The red line in (**b**) exhibits a peak-like shape centered at the Γ point with finite width, suggesting that the $\mathrm{V1} \rightarrow \mathrm{C2}$ transition can induce significant absorption in b-axis polarization near the Γ point. Additionally, due to band dispersion, $\mathrm{V1} \rightarrow \mathrm{C2}$ transitions at points near the Γ point have slightly higher energies than that at the Γ point. Thus, we interpret this as associated with the absorption shoulder observed on the higher energy side of the exciton peak at $\theta = 0°$ in Fig. 1e.

When light polarization is parallel to the a-axis (**c**), two weak TDM peaks are observed for the $\mathrm{V3} \rightarrow \mathrm{C1}$ transition (cyan-color line) on the Γ-X line and the A-Y line. The energy of the latter transition is 2.05 eV, potentially linked to the weak and broad absorption peak-like response observed at ~2 eV in Fig. 1e under a-axis polarization. However, experimental verification of these TDM peaks would require further experimental and theoretical studies.

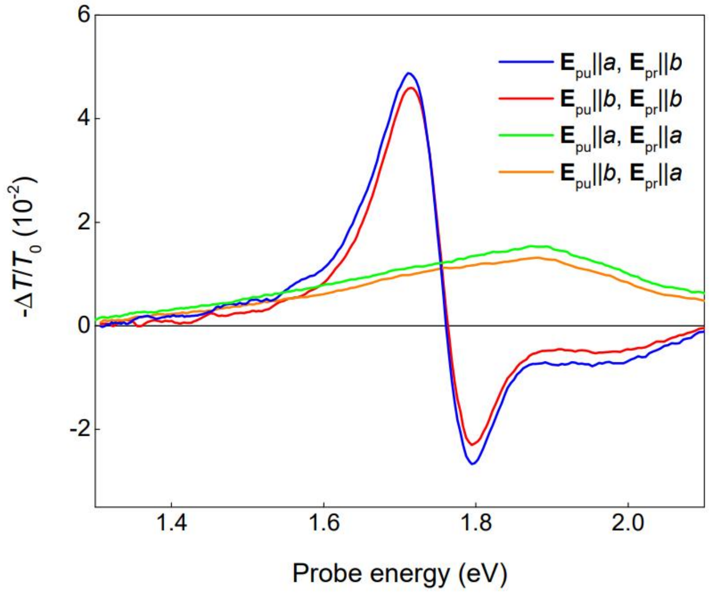

Fig. S2 | Weak dependence of TA on pump polarization.

Transient TA profiles for the $ZrSe_3$ flake sample on a sapphire substrate in Fig. 1d at four different combinations of pump polarization ($\mathbf{E}_{pu}$) and probe polarization ($\mathbf{E}_{pr}$). The pump fluence and energy are fixed at 30 μJ cm^{-2} and 3.1 eV, respectively, and the pump-probe time delay is set to $t = 0.3$ ps. Despite the significant probe dependence of TA, pump polarization has little effect on TA and only leads to slight amplitude changes due to the polarization dependence of pump photon absorption.

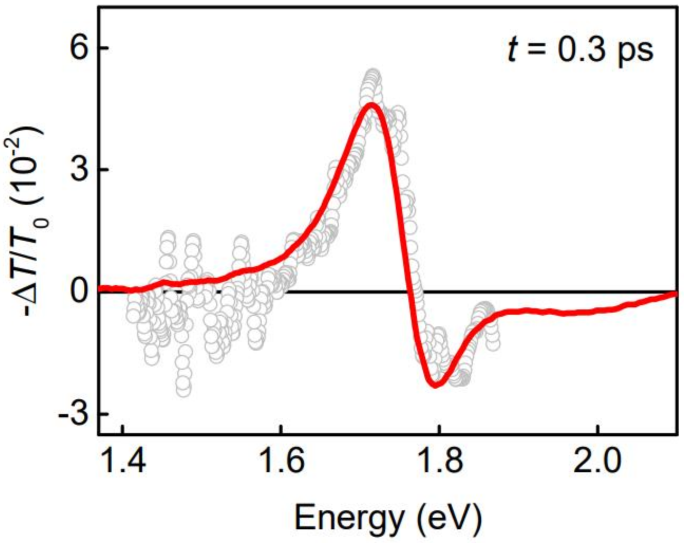

Fig. S3 | Exciton redshift-dominated TA profile.

The red line represents the $-\Delta T/T_0$ profile at $t = 0.3$ ps in Fig. 3a of the main text, where the probe polarization is parallel to the b-axis. In cases where the transient absorption is primarily governed by the exciton shift, the $-\Delta T/T_0$ profile closely follows $-(\log 10)\Delta E_{\mathrm{X}}(\partial A/\partial E)$, where ΔE_{X} denotes the magnitude of the exciton shift, and $\partial A/\partial E$ represents the first derivative of absorbance[2]. The gray circles in this figure plot $-(\log 10)\Delta E_{\mathrm{X}}(\partial A/\partial E)$ spectrum, derived from the steady-state absorbance measured with b-axis polarization. This closely matches the $-\Delta T/T_0$ profile, providing further evidence of the predominance of the redshift. The corresponding shift in the exciton is $\Delta E_{\mathrm{X}} = 25$ meV, comparable to the values observed in other 2D materials[2,3].

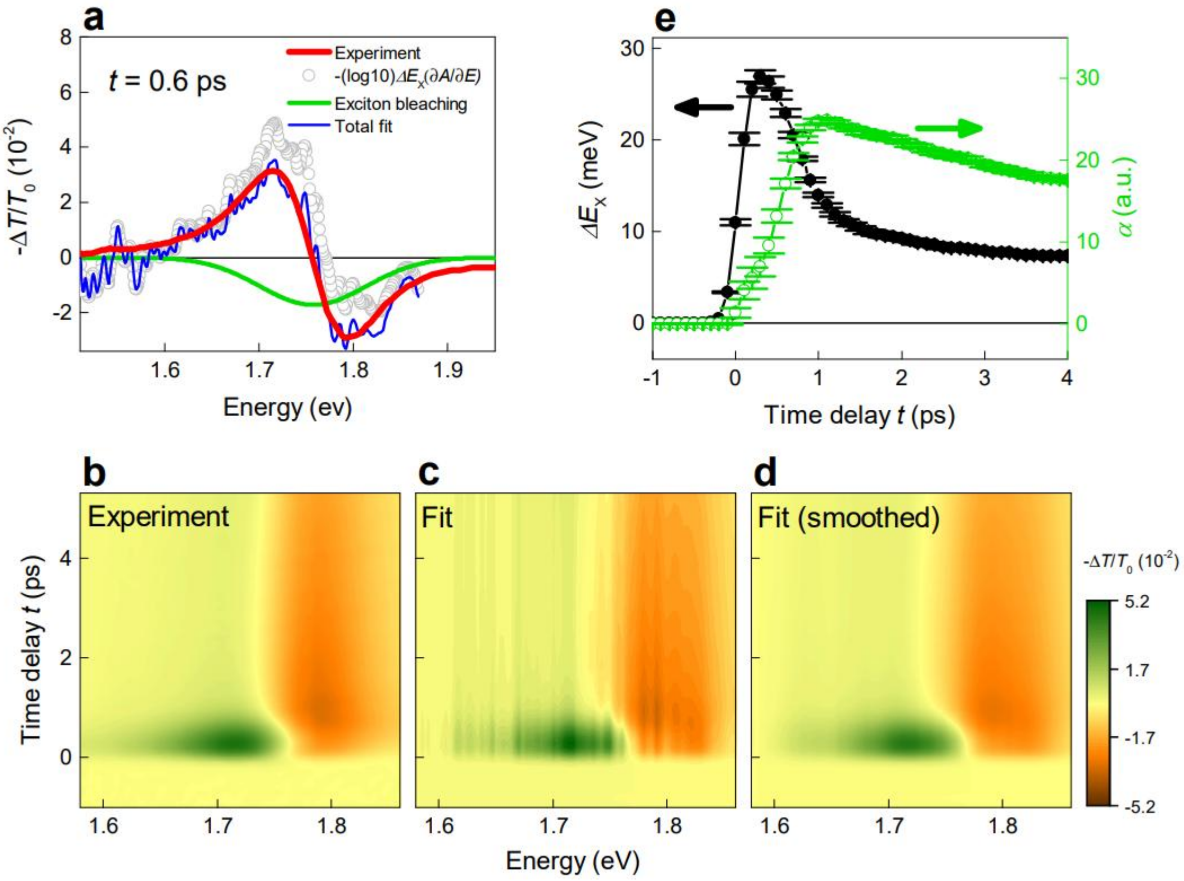

Fig. S4 | Analysis of temporal evolution of TA profile.

In Fig. S3 above, we showed that the $-\Delta T/T_0$ profile at $t = 0.3$ ps closely follows $-(\log 10)\Delta E_{\mathrm{X}}(\partial A/\partial E)$, demonstrating that the redshift of the absorption spectrum dominates the transient response. Here, we analyze the temporal evolution of the $-\Delta T/T_0$ profile. First, (**a**) shows the analysis at a different time point ($t = 0.6$ ps). Here, the red line represents the experimental $-\Delta T/T_0$ profile, and the gray circles represent $-(\log 10)\Delta E_{\mathrm{X}}(\partial A/\partial E)$. Unlike the analysis at $t = 0.3$ ps in Fig. S3, $-(\log 10)\Delta E_{\mathrm{X}}(\partial A/\partial E)$ alone cannot reproduce the $-\Delta T/T_0$ spectrum at $t = 0.6$ ps. This is because carriers relaxed to the band edge cause exciton bleaching via state filling, which significantly affects the $-\Delta T/T_0$ profile. To account for the bleaching effect, we introduced a phenomenological Gaussian bleaching function, $G(E) = -\alpha \exp\left(-\left(\frac{E-(E_0-\Delta E_{\mathrm{X}})}{w}\right)^2\right)$, as shown by the green line in (**a**), where the Gaussian amplitude, α, denotes the degree of exciton bleaching, and w is the width. The Gaussian center is $E_0 - \Delta E_{\mathrm{X}}$, where $E_0 = 1.78$ eV is the position of the steady-state absorption peak in Fig. 1e, and ΔE_{X} is the magnitude of the exciton redshift. Therefore, the total fit function is as follows:

$$-\frac{\Delta T}{T_0}(E) = -(\log 10)\Delta E_{\mathrm{X}}\left(\frac{\partial A}{\partial E}\right) - \alpha \exp\left(-\left(\frac{E-(E_0-\Delta E_X)}{w}\right)^2\right) \quad \text{(S1)}$$

The resulting fit is shown by the blue line in (**a**), which well reproduces the $-\Delta T/T_0$ profile.

This fitting method was applied to other time points to reproduce the temporal evolution of the $-\Delta T/T_0$ profile. Only two free parameters, ΔE_{X} and α, were used, with the Gaussian width fixed at $w = 0.08$ eV for simplicity. (**b**) and (**c**) show the experimentally measured data (taken from Fig. 2a in the main text) and the corresponding fitting result, respectively. Due to the relatively high noise in the experimental $\partial A/\partial E$ curve used for fitting, the fit in (**c**) exhibits an oscillative feature. Therefore, for a clearer comparison with the experimental data, we smoothed the fit curves in (**c**) using the adjacent-average method, as shown in (**d**), which well reproduces the experimental data in (**b**).

The temporal evolution of the fitting parameters is presented in (**e**). Here, ΔE_{X} (black dots, left axis) shows rapid dynamics within 1 ps, which aligns well with our interpretation that the observed sub-picosecond PA1 response arises from the exciton redshift and subsequent its rapid mitigation. Additionally, α (green circles, right axis), corresponding to exciton bleaching, exhibits a relatively slow rise, reflecting the delayed buildup of exciton bleaching as the pump-generated carriers cool down to the band edge, as proposed in the main text.

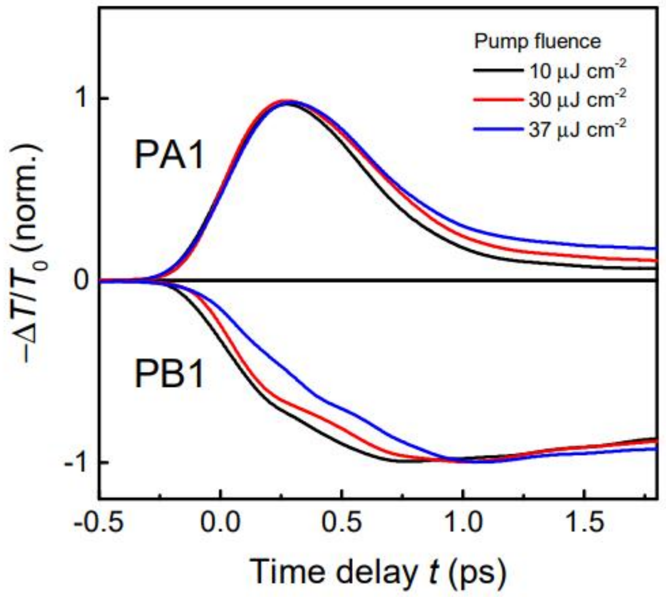

Fig. S5 | Pump fluence-dependent PA1 and PB1 dynamics.

This figure shows normalized $-\Delta T/T_0$ traces measured at peaks PA1 and PB1 when the probe polarization was parallel to the b-axis, for three different pump fluences. Other experimental conditions are the same as those in Fig. 3 of the main text. Both the decay of PA1 and the rise of PB1 are delayed as the pump fluence increases. This trend agrees with the typical characteristics of carrier cooling dynamics and suggests a possible hot phonon bottleneck effect[4–6]. This is consistent with our interpretation that the sub-picosecond decay of PA1 originates from hot carrier cooling.

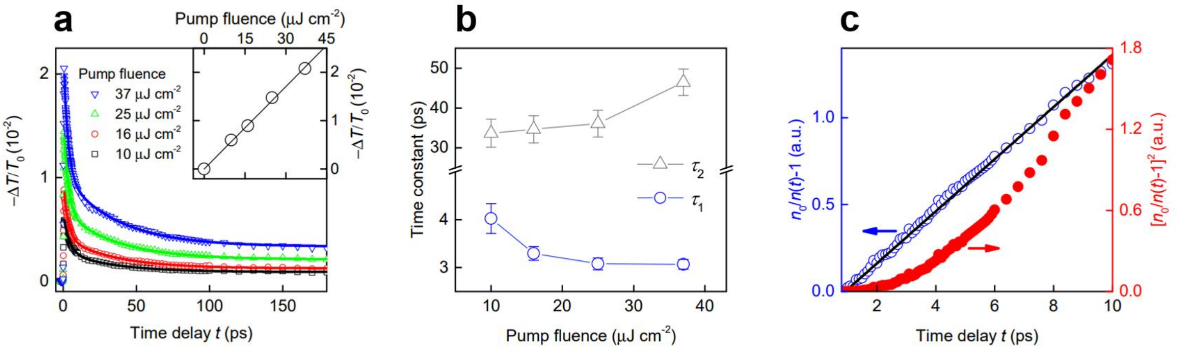

Fig. S6 | Analysis of PA2 decay dynamics.

a-c Symbols in (**a**) present pump fluence-dependent $-\Delta T/T_0$ traces recorded at the peak of PA2 (1.88 eV) with probe polarization parallel to the a-axis. The pump polarization is held fixed along the b-axis. Their peak values near zero-time delay are depicted in the inset of (**a**), showing a linear dependence on pump fluence. The traces in panel (**a**) exhibit biexponential decay, as indicated by the solid-line fits. Corresponding time constants τ_1 = ~3-4 ps and τ_2 = ~30-50 ps are presented in (**b**). We further discuss the potential origins of these decay components.

First, τ_1 decreases with increasing pump fluence, which is a characteristic behavior of Auger-type recombination. In 2D materials, two-body processes such as exciton-exciton annihilation and defect-assisted Auger recombination, as well as three-body processes like three-carrier Auger recombination, are typically observed. The carrier density dynamics ($n(t)$) in two-body and three-body processes are described by the following equations, respectively[7]:

$$[n_0/n(t) - 1] = C_2 n_0 t \qquad \text{(two-body)} \qquad \text{(S2)}$$

$$[n_0/n(t) - 1]^2 = C_3 n_0 t \qquad \text{(three-body)} \qquad \text{(S3)}$$

Here, n_0 represents the initial carrier density, and C_2 (C_3) denotes the two-body (three-body) Auger coefficient. To determine the dominant process among these, we assume that the measured TA traces are proportional to carrier density dynamics (i.e., $-\Delta T/T_0 \propto n(t)$), and obtained the traces of $[n_0/n(t) - 1]$ and $[n_0/n(t) - 1]^2$ from the measured $-\Delta T/T_0$ for 16 μJ cm^{-2} in (A). The resulting $[n_0/n(t) - 1]$ and $[n_0/n(t) - 1]^2$ traces are presented in (**c**) as blue circles (left axis) and red dots (right axis), respectively. We can see that $[n_0/n(t) - 1]$ is linearly proportional to t, indicating the dominance of the two-body Auger process. Using equation (S2), the slope of the linear fit for $[n_0/n(t) - 1]$ (black line in (**c**)) and the initial density of $n_0 \approx 2.1 \times 10^{13}$ cm^{-2} yield the Auger coefficient of $C_2 \approx 7.1 \times$

10^{-3} cm^2 s^{-1}. This value is of the same order of magnitude as the two-body Auger recombination coefficients observed in other 2D layered materials[8,9].

In contrast to τ_1, τ_2 dynamics exhibits a slowing trend with increasing pump fluence in panel (**b**). Such dynamics can be attributed to carrier trapping by defects and saturation of defect sites at high carrier densities[8]. While carrier trapping by defects in 2D layered materials often occurs within a short time scale of ~1 ps[10], trapping timescales can be delayed to the range of 10s-100s ps when defect states are located deep within the band gap[11,12]. Therefore, the observed τ_2 dynamics on the order of ~30-50 ps may be interpreted as carrier trapping by deep defect levels. However, to confirm such attribution, more precise studies on the defect levels of $ZrSe_3$ are required.

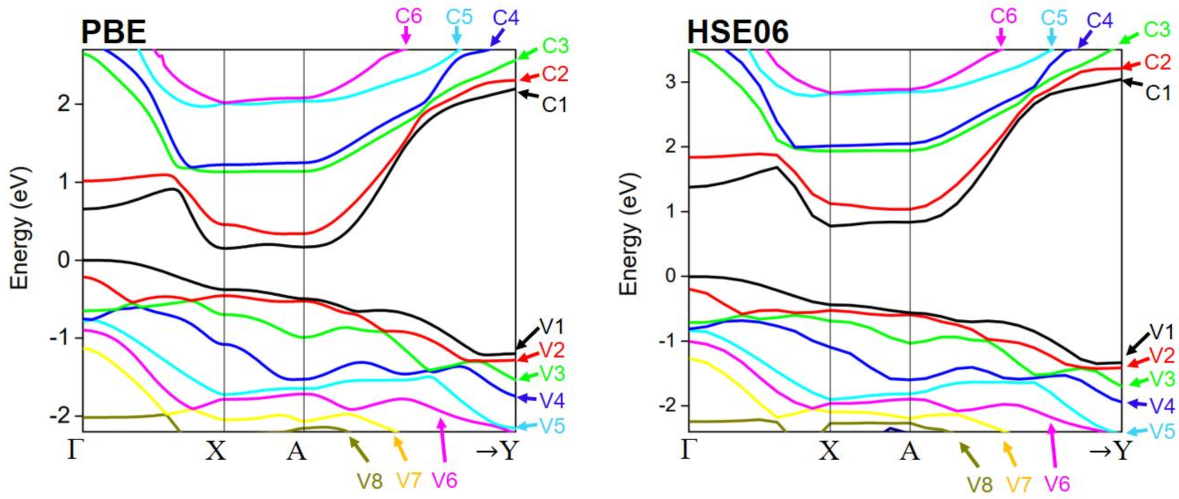

Fig. S7 | Theoretical calculation of band structure.

The band structures of bulk $ZrSe_3$ calculated by the PBE and HSE06 methods are shown, with the conduction bands labeled as C1, C2, etc., and the valence bands labeled as V1, V2, etc. The PBE structure exhibits a significantly underestimated fundamental bandgap compared to the HSE06 structure, a well-known characteristic of PBE calculations[13]. However, there is not a significant dependence on the computational method for the spacings between conduction bands and between valence bands. Therefore, the transition energies in Fig. 4b do not exhibit significant differences between PBE and HSE06.

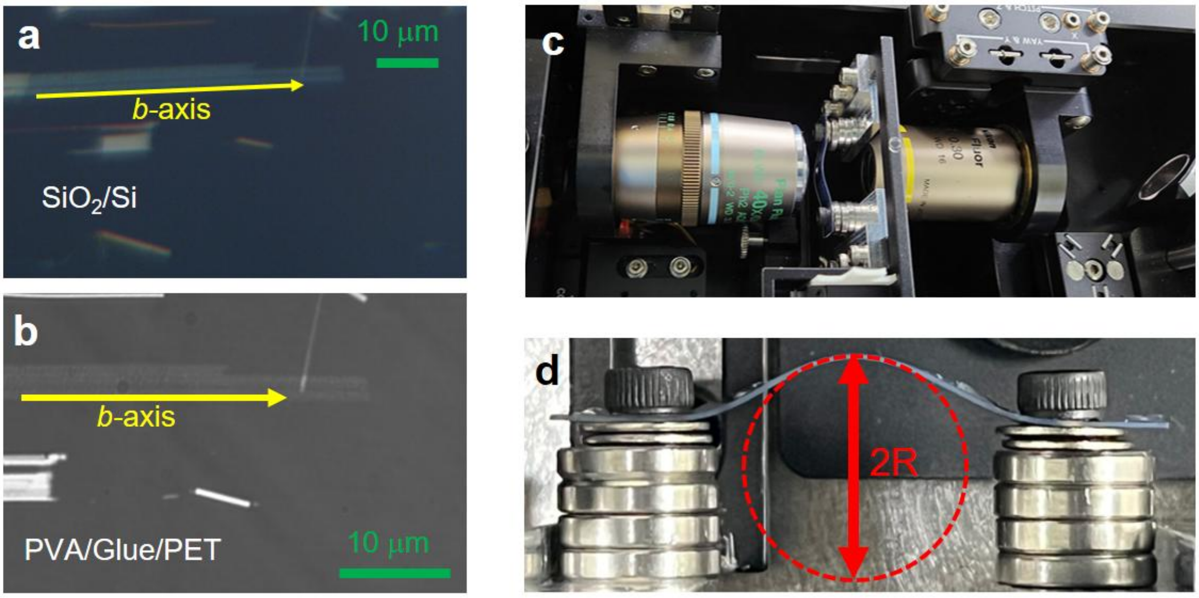

Fig. S8 | Details on strain-dependent ultrafast TA microscopy.

a For the strain-dependent TA experiment, PVA-coated $ZrSe_3$ sample flakes were prepared on flexible PET substrates using the method developed by Li *et al.*[14]. In this method, the PVA coating plays a crucial role in preventing the flake from slipping during substrate bending.[14] Initially, a commercially sourced $ZrSe_3$ bulk crystal was mechanically exfoliated with Scotch tape and transferred to a polydimethylsiloxane (PDMS) film. These exfoliated flakes were then transferred onto a cleaned 5 cm diameter SiO_2/Si substrate. The sample $ZrSe_3$ flake was identified using an optical microscope, as shown in panel (**a**). The sample flake is indicated by the b-axis arrow. The height of the flake measured by AFM was ~31 nm. **b,c** Then, the $ZrSe_3$ flake was then spin-coated with a 10% PVA solution on the SiO_2/Si substrate (at 1000 rpm for 40 seconds) and heated on a hot plate at 70°C for 30 seconds to solidify the PVA layer. Superglue was applied to the PVA layer of the PVA/$ZrSe_3/SiO_2/Si$ composite, following which a 0.3 mm thick PET film was attached to this glue layer. Once dry, this process resulted in the formation of a layered structure comprising PET/glue/PVA/ $ZrSe_3$ / SiO_2 / Si. Then, the PET/glue/PVA/ $ZrSe_3$ layer was peeled from the SiO_2 / Si substrate with tweezers. The position of the $ZrSe_3$ flake within this layer was confirmed under an optical microscope, as shown in panel (**b**). The PET/glue/PVA/$ZrSe_3$ layer was carefully cut into a 3.8 cm by 1.0 cm rectangle, ensuring the flake at its center was aligned such that its b-axis was parallel to the long axis of the rectangle. It was then mounted on a bending stage, and subsequently inserted into the TA microscope module, as shown in panel (**c**). **d** The direction of applied strain was aligned as parallel as possible with the flake's b-axis. Strain (ε) was estimated using the

formula $\varepsilon = \tau/R$, where R is the radius of the curved substrate (indicated in panel (**d**)), and 2τ is the thickness of the PET/glue/PVA composite, measured with a digital thickness gauge[14].

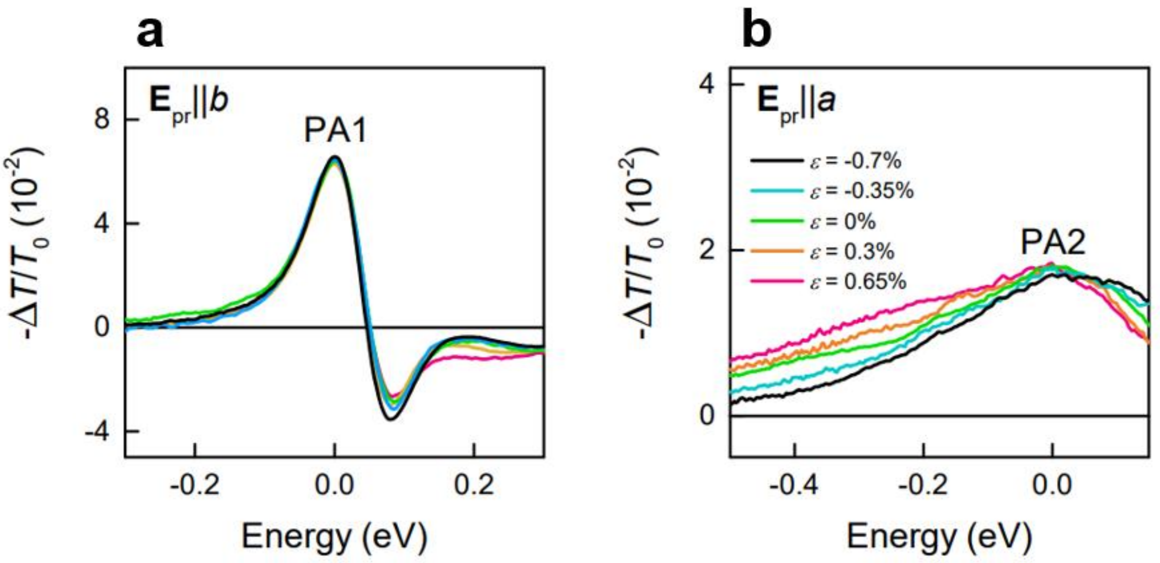

Fig. S9 | Strain dependence of TA profiles.

a First, we adjusted the $-\Delta T/T_0$ spectra at $\mathbf{E}_{\mathrm{pr}}||b$ from Fig. 5d in the main text, aligning the PA1 peak position at 0 eV. The resulting curves in (**a**) reveal that the peak amplitude and width of PA1 remain relatively stable under strain, consistent with prior steady-state reflection measurements on $ZrSe_3$ excitons[15]. This result suggests that within our strain range, the exciton's peak position undergoes the most significant changes (Fig. 5d), while its overall line shape remains largely unchanged.

b Next, we adjusted the $-\Delta T/T_0$ spectra at $\mathbf{E}_{\mathrm{pr}}||a$ from Fig. 5d, aligning the PA2 peak position at 0 eV. The peak amplitude weakly depends on strain, whereas the spectral shape asymmetry varies with strain. At maximum compressive strain ($\varepsilon = -0.7\%$), the $-\Delta T/T_0$ profile exhibits an asymmetric line-shape with the higher side of the center being slightly larger than the left side of the center. But as the strain shifts to tensile, the asymmetric shape changes so that the low energy side becomes pronounced. Such spectral shape variations partially influence the anisotropy ratio of polarization-dependent optical switching in Fig. 5e, although the primary factor is the peak shifts in PA1 and PA2.

The strain-dependent asymmetry of the PA2 peak can arise from various factors. First, the $\mathrm{C1} \rightarrow \mathrm{C6}$ excited state absorption responsible for PA2 is influenced by the dispersion of the C1 and C6 bands and their strain-dependent variations, impacting the spectral shape. In Fig. 6a of the main text, we observed relatively large changes in the C1 band by strain, which may be associated with the strain dependence of the spectral shape in panel (**b**). Second, asymmetric absorption resonances are commonly explained by Fano-type interference, arising from

interference between a single transition and continuous transitions[16]. However, verifying Fano interference requires more precise line-shape studies. Rather than such line-shape analyses, our study focuses on the theoretical analysis of the shift in the PA2 position due to strain, as shown in Fig. 6a, d of the main text.

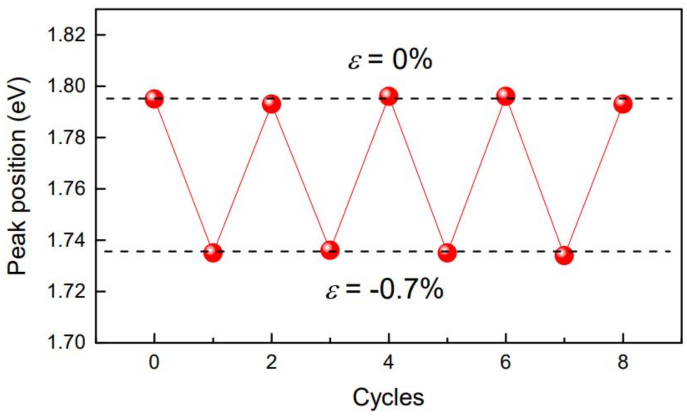

Fig. S10 | Multiple strain-relaxation test.

Red dots represent the peak positions of PA2 measured across multiple compressive strain-relaxation cycles. Both pump and probe polarizations were set in the b-axis direction.

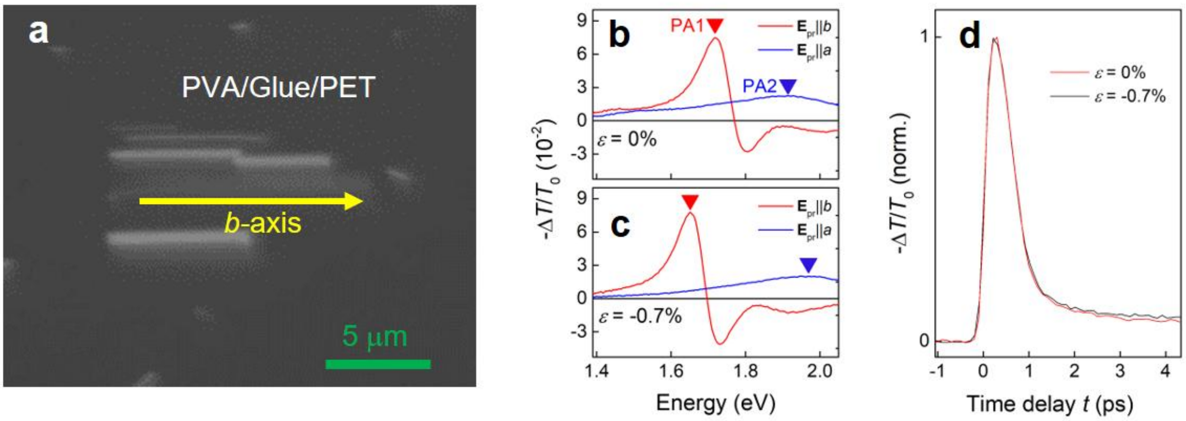

Fig. S11 | Strain-controlled optical switching after 20 days of air exposure.

a We have tested the long-term stability of the strain-dependent optical switching. To this end, we fabricated a PVA-coated 39-nm-thick ZrSe3 adhered to a PET substrate, as shown by the optical image in this panel. After exposing this sample to air for 20 days, we conducted ultrafast transient absorption (TA) experiments.

b,c The resulting probe polarization-resolved transient $-\Delta T/T_0$ profiles at $\varepsilon = 0\%$ (unstrained) and $\varepsilon = -0.7\%$ (compressive strained) are shown in (**b**) and (**c**), respectively. The pump energy and fluence were set to 3.1 eV and 30 μJ cm^{-2}, respectively, with the polarization fixed along the b-axis. The pump-probe time delay was fixed at t = 0.3 ps, where the sub-picosecond PA1 dynamics show maximum $-\Delta T/T_0$ amplitudes. The PA1 peak in the $\mathbf{E}_{\mathrm{pr}}||b$ polarization redshifts by ~67 meV due to compressive strain (indicated by red triangles), while the PA2 peak in the $\mathbf{E}_{\mathrm{pr}}||a$ polarization blueshifts by ~53 meV (indicated by blue triangles). As a result, the anisotropic ratio of the $-\Delta T/T_0$ signal at the PA1 peak position increases from $\rho \approx 5.0$ to $\rho \approx 10.5$ as the spectral positions of PA1 and PA2 move apart by compressive strain.

d Normalized $-\Delta T/T_0$ traces at PA1 for $\varepsilon = 0\%$ and $\varepsilon = -0.7\%$ are shown. Both traces exhibit sub-picosecond switching dynamics.

All these results are in agreement with those presented in Fig. 5 of the main text, demonstrating high stability under air exposure. This aligns well with previous findings on the excellent strain tunability of $ZrSe_3$ over several months of air exposure[15].

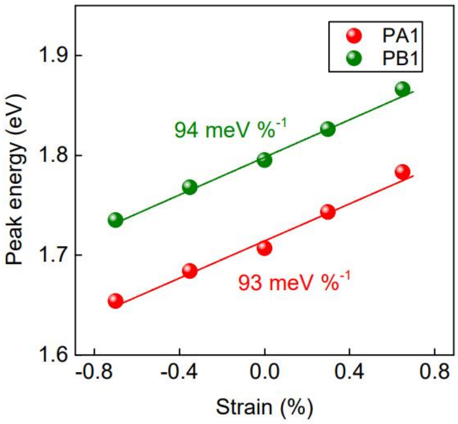

Fig. S12 | Strain-dependent peak shift rate.

Dots represent strain-dependent peak positions of PA1 and PB1, obtained from the blue lines in Fig. 5d. Their shift rates derived from linear fittings are nearly identical, at 93 $\mathrm{meV}\ \%^{-1}$ and 94 $\mathrm{meV}\ \%^{-1}$, respectively. This result indicates that the exciton is shifted due to strain, rather than the pump excitation.

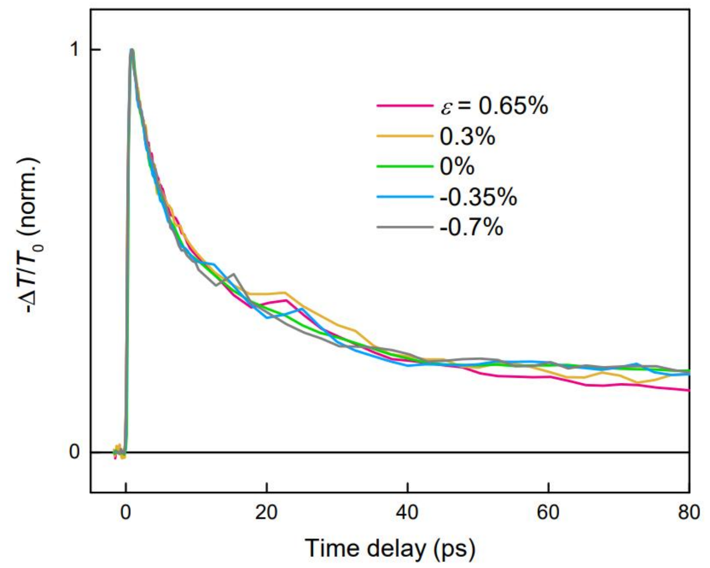

Fig. S13 | Strain-dependent TA dynamics of PA2.

The solid lines depict strain-dependent normalized $-\Delta T/T_0$ traces at the PA2 peak measured with probe polarization parallel to the a-axis, showing minimal dependence on strain.

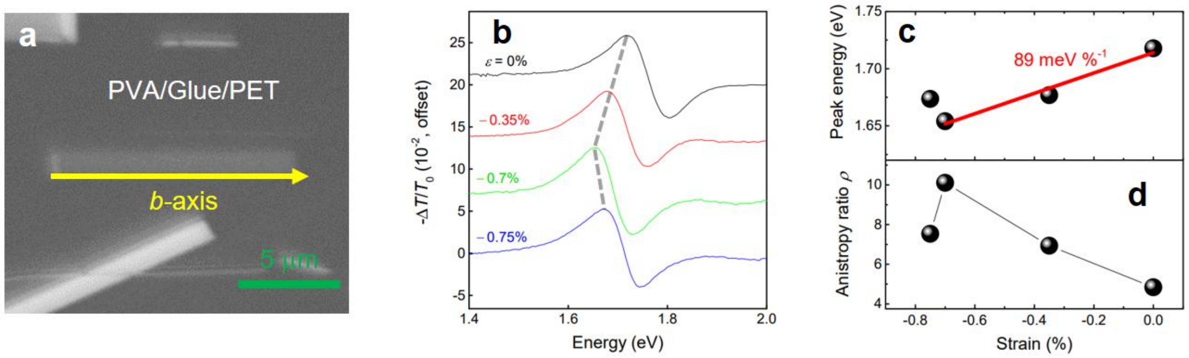

Fig. S14 | The limits of polarization selectivity induced by compressive strain.

a We have investigated the limitations of the polarization selectivity induced by compressive strain. This panel shows an optical image of the sample prepared for this investigation: a ~23-nm-thick $ZrSe_3$ flake coated with PVA and bonded to a PET substrate.

b,c Panel **(b)** shows compressive strain-dependent $-\Delta T/T_0$ profiles of the sample in (**a**) at the $\mathbf{E}_{\text{pr}}||b$ polarization. Offsets were used for clarity. The time delay was fixed at $t = 0.3$ ps. The pump energy and fluence were set to 3.1 eV and 30 μJ cm^{-2}, respectively, with the pump polarization fixed along the b-axis. The peak positions of the PA1 peaks are indicated by the gray dashed line in (**b**), and presented as black dots in (**c**). From 0% to -0.7% strain, the PA1 peak shifts to the lower energy side with increasing compressive strain, and the corresponding shift rate is ~89 meV $\%^{-1}$ (as shown by the red fit line in (**c**)), which agrees with the result in Fig. 5f ($\sim$93 meV $\%^{-1}$). However, when the compressive strain increases to -0.75%, the PA peak shifts back towards the higher energy side. This phenomenon is typically attributed to the buckling of the sample flake caused by compressive strain, leading to delamination from the substrate[17,18]. Thus, the maximum allowable compressive strain is -0.7%, which is comparable to the values observed in recent compressive strain experiments on other 2D materials (-0.7% to -1%)[18].

d This panel shows the corresponding strain-dependent anisotropy ratio (ρ) of $-\Delta T/T_0$ signals for $\mathbf{E}_{\text{pr}}||b$ and $\mathbf{E}_{\text{pr}}||a$ polarizations at the PA1 peak energies. The anisotropy ratio is $\rho \approx 4.8$ at $\varepsilon = 0\%$ and $\rho \approx 10.1$ at $\varepsilon = -0.7\%$, indicating an improvement of ~2.1 times due to compressive strain. However, at $\varepsilon = -0.75\%$, the anisotropy ratio decreases compared to that at $\varepsilon = -0.7\%$, as the compressive strain exceeds the allowable limit. Therefore, combining the result from Fig. 5e and the result presented here, the limit of the anisotropy ratio ranges from ~10 to 11.

Recent studies indicate that the limit of compressive strain applied using flexible substrate bending depends on the presence and method of flake encapsulation[18]. Various encapsulation techniques, including the PVA coating method used in this study[14], have been reported recently[18,19]. Such efforts suggest that the polarization selectivity of optical switching could be further improved in the future.

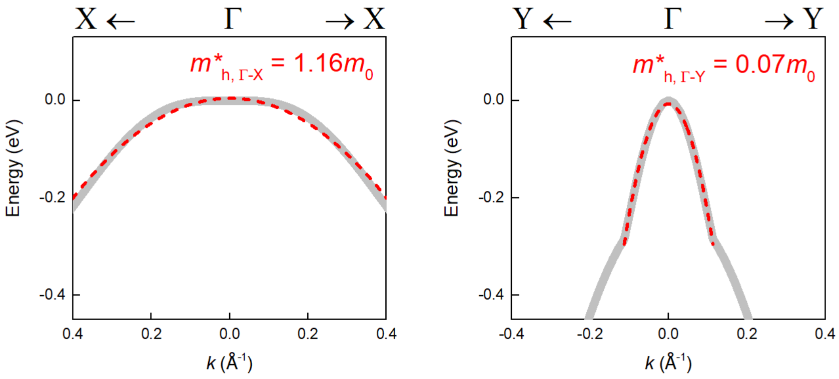

Fig. S15 | Hole effective mass.

The gray lines in both panels show the highest valence band along the $\Gamma - \mathrm{X}$ and $\Gamma - \mathrm{Y}$ directions, respectively. Parabolic fittings (red dashed lines) give the effective masses in each direction, $m^*_{\mathrm{h},\Gamma-\mathrm{X}} \approx 1.16m_0$ and $m^*_{\mathrm{h},\Gamma-\mathrm{Y}} \approx 0.07m_0$, where m_0 is the free electron mass. The geometric mean[20] of these two values is $m^*_{\mathrm{h}} = \sqrt{m^*_{\mathrm{h},\Gamma-\mathrm{X}}\ m^*_{\mathrm{h},\Gamma-\mathrm{Y}}} \approx 0.28m_0$.

Supplementary Note 1. Theoretical calculation details

All first-principles calculations were conducted using the pseudopotential plane wave method implemented in the Vienna Ab-initio Simulation Package[21]. To account for van der Waals interactions, the Grimme method[22] was employed, and a plane-wave cutoff energy of 400 eV was set for all calculations. The geometry optimization of unstrained and strained structures was carried out using the PBE generalized gradient approximation[23] until the Feynman forces on each atom fell below $0.001\ \text{eV Å}^{-1}$. Band structures were calculated using both the PBE and HSE06 hybrid[24] functionals. The polarization-dependent TDMs were obtained from these simulations based on the former functional.

Supplementary Note 2. Quantitative analysis of exciton shift

In the discussion related to Fig. 3 in the main text, we elucidated the sub-picosecond response of PA1 as a result of exciton redshift dynamics. This section quantitatively validates such an interpretation. Initially, immediately following pump excitation in the hot carrier regime (Fig. 3c), the exciton energy shift ($\Delta E_{\text{X}}^{\text{hot}}$) is ascribed to the competition between the BGR-induced bandgap modification (ΔE_{BGR}) and the alteration in exciton binding energy (ΔE_{b}):

$$\Delta E_X^{\text{hot}} = \Delta E_{\text{BGR}} - \Delta E_{\text{b}} \approx -33.4\ \text{meV} \qquad \text{(S4)}$$

Here, the right-hand terms are estimated to be $\Delta E_{\text{BGR}} = -\left(3^{\frac{1}{3}}e/2\pi^{\frac{3}{4}}\epsilon_0\epsilon_r\right)n^{\frac{1}{3}} \approx -53.5\ \text{meV}$ and $\Delta E_{\text{b}} = -\left(3^{\frac{4}{3}}e^2/16\pi^{\frac{3}{4}}\epsilon_0\epsilon_r\right)n^{\frac{1}{3}} \approx -20.1\ \text{meV}$, respectively[25,26], where e denotes the elementary charge, ϵ_0 is the free space permittivity, $\epsilon_r \approx 9$ is the relative permittivity of $ZrSe_3$[27], and $n = 2.1 \times 10^{13}\ \text{cm}^{-2}$ is the density of pump-excited carriers. The exciton shift computed from equation (S4) aligns with the experimentally derived value ($\Delta E_{\text{X}} = -25\ \text{meV}$, Fig. S3). This supports the interpretation that the exciton redshift in the hot carrier regime is determined by the competition between BGR and the exciton binding energy reduction.

As carriers cool to the band edges, holes populating the VBM at the Γ point induce an additional exciton blue shift via the Burstein-Moss (BM) effect (Fig. 3d). This shift is estimated at $\Delta E_{\text{BM}} = \left(h^2 3^{\frac{2}{3}}e/2\pi^{\frac{2}{3}}m_h^*\right)n^{\frac{2}{3}} \approx 37.6\ \text{meV}$,[28] where h is the Planck constant, $m_h^* = 0.28m_0$ is the geometric mean of the hole effective mass (Fig. S15), and m_0 is the free

electron mass. Consequently, the exciton shift in the cold carrier regime,

$$\Delta E_{\mathrm{X}}^{\mathrm{cold}} = \Delta E_{\mathrm{BGR}} - \Delta E_{\mathrm{b}} + \Delta E_{\mathrm{BM}} \approx -4.2\ \mathrm{meV} \qquad \text{(S5)}$$

, is markedly small, suggesting that the initial redshift from BGR is almost canceled out by the BM effect. This conclusion aligns with PA1's rapid diminution on the sub-picosecond scale due to carrier cooling, consistent with the observation in Fig. 3a.

However, these calculations have certain limitations. First, in calculating changes to the exciton binding energy, we only considered carrier density, but lattice and carrier temperatures could also affect it[29]. Second, our calculations assumed that the carrier density produced by the pump remains unchanged during the cooling process. This assumption is based on the fact that the timescale of carrier population dynamics is much longer than the cooling (Fig. S6b), spanning several picoseconds to tens of picoseconds. However, there could be fast depopulation mechanisms, such as carrier trapping by shallow defects, that occur on similar timescales as hot carrier cooling. Third, as pump-created holes and electrons relax to the band edges at the Γ and X points, respectively, they might form momentum-indirect excitons. These possible indirect excitons could further contribute to changes in the energy of probe-generated excitons via Coulomb interactions[30]. Determining the contributions of these additional factors requires further experimental and theoretical research, extending beyond the scope of this study, which primarily focuses on the polarization dependence and strain tuning of ultrafast switching.

Supplementary references

1. El Alaoui Lamrani, H. & Aubin, M. Elliott's model and thermoreflectivity: Application to the layered structure $ZrSe_3$. *Phys. Rev. B* **43**, 4827–4834 (1991).
2. Bae, S. *et al.* Exciton-dominated ultrafast optical response in atomically thin $PtSe_2$. *Small* **17**, 2103400 (2021).
3. Sie, E. J. *et al.* Observation of exciton redshift-blueshift crossover in monolayer WS_2. *Nano Lett.* **17**, 4210–4216 (2017).
4. Nie, Z. *et al.* Ultrafast carrier thermalization and cooling dynamics in few-layer MoS_2. *ACS Nano* **8**, 10931–10940 (2014).
5. Yang, Y. *et al.* Observation of a hot-phonon bottleneck in lead-iodide perovskites. *Nat. Photonics* **10**, 53–59 (2016).
6. Suk, S. H. *et al.* Polarization-driven ultrafast optical switching in TiS_3 nanoribbons via anisotropic hot carrier dynamics. *Adv. Opt. Mater.* **11**, 2300370 (2023).
7. Robel, I., Bunker, B. A., Kamat, P. V. & Kuno, M. Exciton recombination dynamics in CdSe nanowires: Bimolecular to three-carrier Auger kinetics. *Nano Lett.* **6**, 1344–1349

(2006).

8. Shin, H. J., Bae, S. & Sim, S. Ultrafast Auger process in few-layer $PtSe_2$. *Nanoscale* **12**, 22185–22191 (2020).

9. Sim, S. *et al.* Role of weak interlayer coupling in ultrafast exciton-exciton annihilation in two-dimensional rhenium dichalcogenides. *Phys. Rev. B* **101**, 174309 (2020).

10. Gao, L., Hu, Z., Lu, J., Liu, H. & Ni, Z. Defect-related dynamics of photoexcited carriers in 2D transition metal dichalcogenides. *Phys. Chem. Chem. Phys.* **23**, 8222–8235 (2021).

11. Wang, H., Zhang, C. & Rana, F. Ultrafast dynamics of defect-assisted electron-hole recombination in monolayer MoS_2. *Nano Lett.* **15**, 339–345 (2015).

12. Wang, L. *et al.* Auger-type process in ultrathin ReS_2. *Opt. Mater. Express* **10**, 1092 (2020).

13. Seidl, A., Görling, A., Vogl, P., Majewski, J. & Levy, M. Generalized Kohn-Sham schemes and the band-gap problem. *Phys. Rev. B - Condens. Matter Mater. Phys.* **53**, 3764–3774 (1996).

14. Li, Z. *et al.* Efficient strain modulation of 2D materials via polymer encapsulation. *Nat. Commun.* **11**, 1151 (2020).

15. Li, H. *et al.* Strongly anisotropic strain-tunability of excitons in exfoliated $ZrSe_3$. *Adv. Mater.* **34**, 2103571 (2022).

16. Miroshnichenko, A. E., Flach, S. & Kivshar, Y. S. Fano resonances in nanoscale structures. *Rev. Mod. Phys.* **82**, 2257–2298 (2010).

17. Brennan, C. J., Nguyen, J., Yu, E. T. & Lu, N. Interface adhesion between 2D materials and elastomers measured by buckle delaminations. *Adv. Mater. Interfaces* **2**, 1500176 (2015).

18. Li, H. *et al.* Towards efficient strain engineering of 2D materials: A four-points bending approach for compressive strain. *Nano Res.* **17**, 5317–5325 (2024).

19. Jin, Y. *et al.* Stretching graphene to 3.3% strain using formvar-reinforced flexible substrate. *Exp. Mech.* **62**, 761–767 (2022).

20. Xi, J., Zhao, T., Wang, D. & Shuai, Z. Tunable electronic properties of two-dimensional transition metal dichalcogenide alloys: a first-principles prediction. *J. Phys. Chem. Lett.* **5**, 285–291 (2014).

21. Kresse, G. & Furthmüller, J. Efficiency of *ab-initio* total energy calculations for metals and semiconductors using a plane-wave basis set. *Comput. Mater. Sci.* **6**, 15–50 (1996).

22. Grimme, S., Antony, J., Ehrlich, S. & Krieg, H. A consistent and accurate *ab initio* parametrization of density functional dispersion correction (DFT-D) for the 94 elements H-Pu. *J. Chem. Phys.* **132**, 154104 (2010).

23. Perdew, J. P., Burke, K. & Ernzerhof, M. Generalized gradient approximation made simple. *Phys. Rev. Lett.* **77**, 3865 (1996).

24. Krukau, A. V., Vydrov, O. A., Izmaylov, A. F. & Scuseria, G. E. Influence of the exchange screening parameter on the performance of screened hybrid functionals. *J. Chem. Phys.* **125**, 224106 (2006).

25. Fan, K., Chan, C. C. S., Yuan, L., Yan, K. & Wong, K. S. New insights into hot-charge relaxation in lead halide perovskite: dynamical bandgap change, hot-biexciton effect, and photo-bleaching shift. *ACS Photonics* **9**, 2304–2314 (2022).

26. Sun, Q. C. *et al.* Observation of a burstein-moss shift in rhenium-doped MoS_2 nanoparticles. *ACS Nano* **7**, 3506–3511 (2013).

27. Khumalo, F. S., Olson, C. G. & Lynch, D. W. Optical dichroism in $ZrSe_3$ layer-type materials. *Phys. B+C* **105**, 163–168 (1981).

28. Feneberg, M. *et al.* Band gap renormalization and Burstein-Moss effect in silicon- and germanium-doped wurtzite GaN up to 1020 cm^{-3}. *Phys. Rev. B* **90**, 075203 (2014).

29. Ruf, F. *et al.* Temperature-dependent studies of exciton binding energy and phase-transition suppression in $(Cs,FA,MA)Pb(I,Br)_3$ perovskites. *APL Mater.* **7**, 031113 (2019).

30. Bobrysheva, A. I. The interaction of two excitons in a crystal. *Phys. Stat. Sol.* **16**, 337 (1966).
